# Viral Oncology: Molecular Biology and Pathogenesis

**DOI:** 10.3390/jcm6120111

**Published:** 2017-11-29

**Authors:** Uyen Ngoc Mui, Christopher T. Haley, Stephen K. Tyring

**Affiliations:** 1 Center for Clinical Studies, Houston, TX 77004, USA; fellowwebster@ccstexas.com (C.T.H.); styring@ccstexas.com (S.K.T.); 2 Department of Dermatology, University of Texas Health Science Center at Houston, Houston, TX 77004, USA

**Keywords:** viral oncology, human oncovirus, EBV, HPV, HBV, HCV, HTLV-1, HHV-8, MCPyV

## Abstract

Oncoviruses are implicated in approximately 12% of all human cancers. A large number of the world’s population harbors at least one of these oncoviruses, but only a small proportion of these individuals go on to develop cancer. The interplay between host and viral factors is a complex process that works together to create a microenvironment conducive to oncogenesis. In this review, the molecular biology and oncogenic pathways of established human oncoviruses will be discussed. Currently, there are seven recognized human oncoviruses, which include Epstein-Barr Virus (EBV), Human Papillomavirus (HPV), Hepatitis B and C viruses (HBV and HCV), Human T-cell lymphotropic virus-1 (HTLV-1), Human Herpesvirus-8 (HHV-8), and Merkel Cell Polyomavirus (MCPyV). Available and emerging therapies for these oncoviruses will be mentioned.

## 1. Introduction

In 1964, the first human oncovirus was discovered, when Epstein-Barr virus (EBV) was detected in Burkitt lymphoma cells by electron microscopy [1]. This finding built upon the landmark avian cancer virus research performed by Rous in the early 20th century [2,3]. Viral oncology knowledge and cancer surveillance have grown immensely since then. Approximately 20% of all cancers are associated with infectious agents [4], and 12% of all cancers are caused by oncoviruses [5,6,7]. 80% of viral cancers occur in the developing world [5,6,7].

Oncovirus infections are common, but these infections rarely result in cancer [4]. One or more additional insults, such as chronic inflammation, environmental mutagens, or immunosuppression, are required for cancer development [4,5]. Additionally, viruses are only an absolute requirement for oncogenesis in Kaposi sarcoma and cervical cancer [4,8]. Oncoviruses are classified as direct or indirect carcinogens, although some overlap exists between the distinctions [8]. Direct carcinogenic viruses possess viral oncogenes that directly contribute to neoplastic cellular transformation, whereas indirect carcinogens cause chronic inflammation, which can lead to oncogenic transformation [9,10]. Oncogenic DNA viruses include EBV, hepatitis B virus (HBV), human papillomavirus (HPV), human herpesvirus-8 (HHV-8), and Merkel cell polyomavirus (MCPyV). Oncogenic RNA viruses include, hepatitis C virus (HCV) and human T-cell lymphotropic virus-1 (HTLV-1).

Viral cancers do not arise acutely after infection, but instead develop 15–40 years later [4]. One exception is a rare EBV-associated lymphoproliferative disease, which can occur shortly after infection [4]. In cancers, viral replication is either diminished or absent [4,8], as active replication would lyse the host cell and prevent tumorigenesis. The virus exists intracellularly as naked nucleic acid in the form of a plasmid, episome, or cellular-integrated genome [8]. DNA virus genomes can integrate directly into the host genome, while RNA virus genomes must undergo reverse transcription to DNA before integration can occur [11]. 

In general, all oncoviruses promote tumorigenesis via common pathways. Tumor suppressor pathways, such as p53 and retinoblastoma (Rb), are often inhibited [11,12,13]. Other frequent targets include, tumor necrosis-associated factors (TRAFs) [8], telomerase reverse transcriptase (TERT) [14,15,16,17,18], cytoplasmic PI3K-AKT-mTOR [19], nuclear factor-κB (NF-κB) [20,21,22,23], β-catenin [24], interferon signaling pathways [25], major histocompatibility class-1 (MHC-1), and Janus kinase/signal transducer and activator of transcription (JAK/STAT) [25]. The host DNA damage response pathway (DDR) can also be affected, particularly by DNA viruses [26]. The DDR detects and repairs damaged cellular DNA via responses, initiated by the phosphoinositide-3-like kinase (PIKK) family of serine/threonine kinases, including ataxia-telangiectasia mutated (ATM), ataxia-telangiectasia and RAD3-related (ATR), and DNA-dependent protein kinase (DNA-PK) [27]. Cell cycle progression may be delayed by the DDR until DNA repair is completed or foreign viral DNA is no longer detected [28]. To promote oncogenesis, viral proteins activate aspects of the DDR that are beneficial to viral replication or cellular transformation, such as repair factor recruitment, and inactivate DDR activities that are detrimental to viral DNA survival, such as apoptotic pathways [26]. The oncogenic potential of some viruses has been clearly established, and viruses are thus becoming targets for cancer treatment and prevention [8]. Successful vaccines are already available for HBV and HPV infection prevention [29,30,31]. Antiviral malignancy treatments and therapeutic vaccines have not yet been developed, but are currently under investigation [32].

## 2. Epstein-Barr Virus

Epstein-Barr virus, formerly known as human herpesvirus-4, is one of eight known human viruses belonging to the herpesviridae family. The majority of EBV infection is acquired during childhood via salivary transmission. It is estimated that more than 90% of the world’s population have been infected with EBV by adulthood [33,34]. After primary infection, EBV persists in a latent state, most commonly in resting memory B cells and sometimes in epithelial cells, T cells, or natural killer (NK) cells [33,34,35].

EBV was the first human oncogenic virus discovered, and was originally identified in Burkitt lymphoma cells in 1964 [1,36]. EBV is best known for causing infectious mononucleosis, and has been associated with several malignancies of epithelial and lymphocytic origin. While the association between EBV and B-cell lymphoproliferative disorders has been well-documented, it is now known that EBV can also predispose to NK/T cell lymphoproliferative diseases [33]. 

B-cell lymphoproliferative disorders that have been closely associated with EBV include, Burkitt lymphoma (BL) [37], Hodgkin lymphoma (HL) [38], and post-transplant lymphoproliferative disorder (PTLD) [39]. A wide range of T cell lymphoproliferative disorders have been reported to be EBV associated, including peripheral T cell lymphoma, angioimmunoblastic T cell lymphoma, extranodal nasal type NK/T cell lymphoma, aggressive NK cell leukemia/lymphoma, and cutaneous T-cell lymphoproliferative disorder [34,39]. Epithelial malignancies associated with EBV include gastric carcinoma and nasopharyngeal carcinoma [35].

The mechanism of EBV infection in T and NK cells has not been clearly defined, although positive stains for T and NK cell markers in infectious mononucleosis suggest that primary infection of T and NK cells do occur [40]. It has been proposed that T and NK cells become infected while attempting to kill an EBV-infected cell [41]. Similarly, the pathogenesis of EBV infection in epithelial cells is poorly understood. Since epithelial cells lack CD21 through which EBV enters B lymphocytes, it is believed that EBV gains entry into epithelial cells via direct cell-to-cell contact with infected B lymphocytes [33]. 

### 2.1. EBV Establishes Latency in Resting Memory B Cells

The EBV genome is a linear, double-stranded DNA, measuring approximately 172 kb in length [42]. After entry into B cells, the viral DNA is circularized by joining the terminal repeats and then arranged onto nucleosomes and packaged into a mini-chromosome structure called episome [43]. Post-translational modifications of the episome are important in regulating the shift from lytic to latent replication, and vice versa [43].

EBV enters B lymphocytes via interaction of the EBV surface protein gp350 with the lymphocyte receptor CD21 and HLA class II [44]. During primary infection, EBV replicates in epithelial or B cells of the oropharynx, and then, to escape immune surveillance, turns off most of its genes and enters a state of latency, with resting memory B cells as the primary reservoir [43]. EBV genes expressed during latent infection include six nuclear antigens (EBNA-1, 2, 3A, 3B, 3C, and leader protein (LP)), three latent membrane proteins (LMP-1, 2A, and 2B), two EBV-encoded small RNAs (EBER-1 and 2), and three clusters of microRNAs (miRNAs) [34,43].

There are two models that describe the mechanism by which EBV establishes latency in mature B cells: germinal center model and direct infection model. According to the germinal center model, primary infection occurs in naïve B cells, which proliferate and then enter the germinal center [45]. Once in the germinal center, LMP-1 and LMP-2A provide signals for growth and differentiation into memory B cells by mimicking CD40L-mediated signaling [45]. The memory B cells then exit the germinal center and inactivate gene expression to avoid detection by the immune system [45]. In contrast, the direct infection model proposes that EBV directly infects memory B cells without requiring the sparticipation of the germinal center [43].

**Figure 1:**
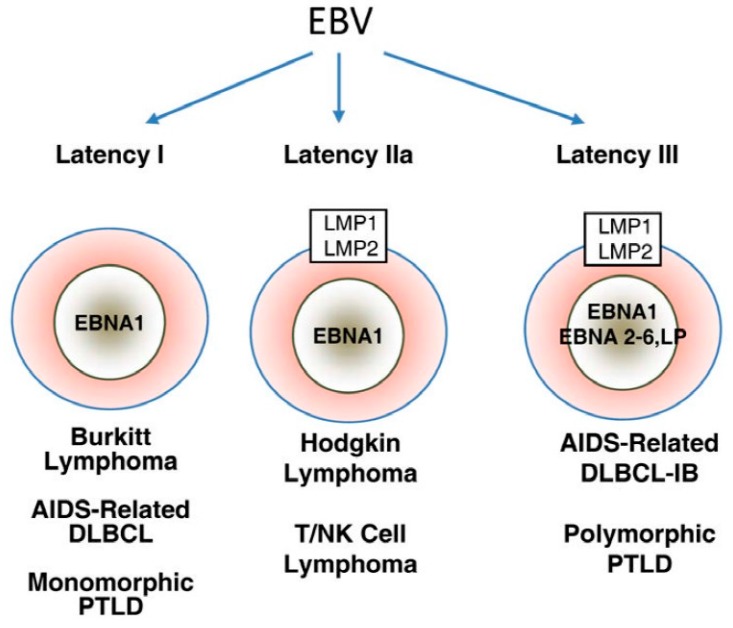
Major patterns of Epstein-Barr virus (EBV) latent gene expression in lymphoproliferative disorders. The main EBV latency patterns and the most common lymphoproliferative disorders in which these patterns are seen are illustrated. Reproduced with permission from Cesarman, E., Gammaherpesvirus and lymphoprolliferative disorders in immunocompromised patients; published by *Cancer Lett.*, 2011.

Three EBV latency programs, latency 0-I, II, and III, have been established based on patterns of latency gene expression in normal B cells (Figure 1) [43]. Latency gene expression is unrestricted in latency III, which is only possible under immune suppressed states as these latency proteins are highly immunogenic [43]. Latency II is restricted to EBNA-1, EBERs, and LMPs [43]. Finally, depending on the phase of the cell cycle, no gene product is expressed in latency 0 or only EBNA-1 is expressed in latency I [46].

Latency III malignancies develop in conditions of impaired T cell immunity, such as HIV infection or transplant-related immunosuppression [43]. EBV-associated large B cell and immunoblastic lymphomas often displa latency III program, regardless of the immune function of the host [47]. In latency II malignancies, such as Hodgkin lymphoma, nasopharyngeal carcinoma, and NK/T cell lymphoma, LMP-1-mediated activation of PI3K/AKT and JAK/STAT pathways is believed to be the main oncogenic event [43]. Finally, whether EBV is a driving or supportive force in latency I malignancies, such as BL, remains to be defined. Most EBV-induced tumor transformation processes require multiple latency proteins, but EBNA-1 is generally thought to be the only viral protein that is expressed in latency I malignancies [48]. Furthermore, Grömminger et al., suggested that EBV plays a supportive role in BL since constitutive *c-myc* activation is the major oncogenic event in all cases of BL, irrespective of EBV status [49]. On the other hand, several studies have found that a minor proportion of these tumors have broader gene expression profile than previously thought, lending support to EBV as the driving force in latency I malignancies.

### 2.2. EBV Oncogenic Proteins

LMP-1 is generally considered as the main oncogenic protein of EBV, and it is essential for the transformation of resting primary B cells into proliferating lymphoblastoid cells [33,34,43]. LMP-1 is a transmembrane protein that acts as a constitutively activated CD40 receptor, leading to activation of downstream signaling pathways involved in the differentiation of memory B lymphocytes and the expression of anti-apoptotic proteins [43]. These downstream signaling pathways include, NF-κB, MAPK/ERK, PI3K/AKT, Notch, and JAK/STAT [50]. The PI3K/AKT and JAK/STAT pathways appear to be the most important pathways in EBV-induced oncogenesis [34,43]. The activation of PI3K/AKT and JAK/STAT pathways contribute to hallmarks of cancer, such as increased genomic instability, apoptosis resistance, limitless replicative potential, reprogramming of energy metabolism, tumor-promoting inflammation, and tissue invasion and metastasis [51]. Furthermore, LMP-1 induces genomic instability through the inhibition of DNA repair mechanisms and suppression of DNA damage checkpoints [34].

LMP-2A enhances cell survival through several mechanisms, such as inhibition of TGF-β1-induced apoptosis [52], upregulation of survivin expression mediated through activation of NF-κB signaling pathway [53], promotion of cyclin E expression, and increase in cell entry into S phase [54]. Furthermore, LMP-2 activates the Lyn/Syk signaling pathway, a tyrosine kinase pathway that is primarily expressed in hematopoietic malignancies that is essential for tumor survival [55,56]. Cells that do not express Syk are more likely to undergo apoptosis [56]. Data also suggest that LMP-2A may activate the Notch signaling pathway, which stimulates cell migration and epithelial-to-mesenchymal transition (EMT) [57]. Moreover, LMP-2A has a unique function of inducing epigenetic changes by promoting STAT3 phosphorylation, leading to the activation of DNA methyltransferases (DNMTs) [58]. 

EBNA-1 is the only viral protein that is expressed in all of the EBV-associated malignancies [33], but understanding of its role in oncogenesis is limited. EBNA-1 is essential for the replication and maintenance of EBV genome, and may act as an oncogene [42]. The promyelocytic leukemia (PML) protein is a tumor suppressor protein that regulates p53 activation [33]. By suppressing PML, EBNA-1 inhibits p53-dependent activation of p21 and apoptosis signaling, which consequently enhances cell survival in spite of DNA damage [33,46]. Furthermore, EBNA-1 protects against apoptosis by downregulating the expression of *myc* oncogene and enhancing the expression of anti-apoptotic proteins Bcl-2 and survivin [34]. In addition, increasing evidence has linked EBNA-1 to the induction of genomic instability [46,59,60]. EBNA-1 activates reactive oxygen species (ROS) production, contributing to chromosomal aberrations [34]. It is postulated that EBNA upregulates NOX2, the catalytic subunit of NADPH oxidase, which is involved in the production of ROS and the subsequent generation of chromosomal aberrations, DNA damage, and telomere abnormalities [46,59,60]. 

EBNA-2 is important for transformed B cell proliferation and the prevention of transformed B cell apoptosis [43]. EBNA-2, in collaboration with EBNA-LP, is directly responsible for initiating the transcription of several viral (LMP-1, LMP-2A) and cellular (MYC, CD21, CD23) proteins that are crucial for B cell immortalization and transformation [43]. Finally, the effects of EBNA-3 are to prevent the accumulation of cyclin-dependent kinase (CDK) inhibitors, to degrade the tumor suppressor protein Rb, to stabilize *c-myc* oncogene, and to suppress pro-apoptotic proteins [61].

Latently, EBV-infected cells express an abundance of viral RNA transcripts, called EBERs, which have been shown to affect various cellular processes, such as cell proliferation, apoptosis, production of growth factors, and cellular signaling [33]. EBERs can alter miRNA expression to repress E-cadherin, which results in EMT [62]. EBERs promote chemoresistance by activating IL-6/STAT3 signaling pathway to downregulate the expression of cell cycle inhibitors p21 and p27 [63]. They also stimulate cell migration through the activation of pro-metastatic molecules pFAK and pPAK1, and the suppression of anti-metastatic molecules RhoGD1 and KAI-1 [33]. EBERs protect cell from apoptosis mediated through IRF-3 and NF-κB signaling and suppression of IFN-α mediated apoptosis [34]. Finally, EBERs induce growth promoting cytokines, such as IL-6, IL-9, IL-10, and IGF-1 [34].

### 2.3. EBV and T Cell Lymphoproliferative Disorders

Although many T cell lymphoproliferative disorders have been linked to EBV infection, understanding of the exact molecular pathogenesis is still limited. The two types of T cell lymphoproliferative disorders in which EBV has the strongest evidence are angioimmunoblastic T cell lymphoma and extranodal nasal type NK/T cell lymphoma [39]. Angioimmunoblastic T cell lymphoma is a subtype of peripheral T cell lymphoma that is characterized by polyclonal expansion of B and T lymphocytes [64]. In angioimmunoblastic T cell lymphoma, EBV is detected primarily in B cells, suggesting that EBV infection of T cell is a secondary event or that viral genome has been lost from the transformed T cells [39].

Extranodal NK/T cell lymphomas predominantly affect the nasal and paranasal sites in 80% of cases [34,65]. The other 20% of cases occur in the skin, soft tissue, gastrointestinal tract, testis, and upper respiratory tract [64,65]. These tumors are rare in Western countries, but are common in East Asia and Latin America [34]. Regardless of the geographical distribution, EBV has been found in more than 90% of all cases [34,64]. The molecular pathogenesis of NK/T cell lymphoma remains to be defined, but the advent of gene expression profiling has elucidated the major signaling pathways contributing to lymphomagenesis [65]. Several gene mutations have been identified in NK/T cell lymphoma, of which *JAK3*, *STAT3*, and *DDX3X* are the major genes involved [65]. As mentioned previously, the JAK/STAT pathway is critical for many cellular processes, and its dysregulation is a major contributor to tumorigenesis. DDX3X is an RNA helicase responsible for initiating RNA translation. Analysis of *DDX3X* mutants showed a decrease in RNA unwinding activity, loss of cell cycle suppression, and transcriptional activation of NF-κB and mitogen-activated protein kinase (MAPK) pathways [65]. These events consequently promote tumor formation.

### 2.4. EBV and Epithelial Malignancies

Gastric carcinoma and nasopharyngeal carcinoma are the most well-established EBV-associated epithelial malignancies. Approximately 10% of gastric cancer worldwide can be attributed to EBV [33]. The prevalence of EBV-positive gastric cancer is higher among males and Caucasian and Hispanic ethnicities [35,66,67]. Malignant transformation often arises from the proximal portion of the stomach, such as cardia and gastric body [66]. 

Nasopharyngeal carcinoma is a rare cancer in most parts of the world, with an incidence less than 1/100,000 in Caucasians to 25–30/100,000 in Chinese per year [68]. The World Health Organization (WHO) categorizes nasopharyngeal carcinoma into three subtypes: keratinizing squamous cell carcinoma (type 1), nonkeratinizing carcinoma (type 2), and undifferentiated carcinoma (type 3) [69]. Types 2 and 3 nasopharyngeal carcinomas are known to be associated with EBV, although the exact mechanism is uncertain [68].

### 2.5. EBV and B-Cell Lymphoproliferative Disorders

The types of B-cell lymphoproliferative disorders associated with EBV infection are PTLD, BL, and HL [37]. According to the WHO classification, PTLDs can be classified into four histological types: (1) early lesions; (2) polymorphic; (3) monomorphic; and, (4) classical Hodgkin lymphoma. Monomorphic PTLD is further classified into B cell neoplasms (Burkitt lymphoma, diffuse large B cell lymphoma, plasma cell myeloma, and plasmacytoma-like lesion) and T cell neoplasms (peripheral T cell lymphoma and hepatosplenic T cell lymphoma) [70].

Early-onset PTLDs are considered EBV-driven polyclonal lymphoproliferations, whereas late-onset PTLDs are true monoclonal diseases that are not necessarily associated with EBV [39]. There are several key pieces of evidence supporting the role of EBV in the pathogenesis of PTLDs: (1) 60–80% of all PTLD cases are EBV-positive, including 90–100% of early-onset PTLDs [71]; (2) monoclonal EBV infection in monomorphic PTLDs supports the hypothesis that the virus has been present since the early stage of tumor cell expansion [39]; (3) development of PTLD is correlated to high EBV viral load and low EBV-specific cytotoxic T lymphocytes (CTLs) [39]; and, (4) treatment with EBV-CTL results in decreased viral load and tumor regression [72].

Burkitt lymphoma can be classified into three distinct variants: endemic (African) BL, sporadic (non-endemic) BL, and immunodeficiency-associated BL. Endemic BL affects the jaw and facial bones of young children in equatorial Africa, whereas sporadic BL involves the gut, upper respiratory tract, or Waldeyer ring in children and young adults, throughout the world [72]. Lymph node and bone marrow are the primary sites of involvement in immunodeficiency-associated BL [72]. Among the subtypes, endemic BL has the strongest association with EBV, where virtually all of the cases are EBV-positive [39]. In contrast, only 30% of sporadic and 25–40% of immunodeficiency-associated BL are EBV-positive [37]. 

Hodgkin lymphoma originates from B cells that have acquired mutations leading to the aberrant expression of cellular markers. Approximately 40–50% of cases of HL in the United States (U.S.) are EBV-positive [38]. Among the variants of HL, EBV is found in 95% of lymphocyte-depleted HL, 70% of mixed-cellularity HL, 10–40% of nodular sclerosing HL, and most cases of immunodeficiency-associated HL [72]. In contrast, nodular lymphocyte predominant HL is generally EBV-negative [72]. The survival of Hodgkin’s Reed-Sternberg (HRS) cells, the characteristic transformed B cells in HL, is partly dependent on NF-κB activation. One mechanism that is responsible for the activation of NF-κB is the binding of CD40L to CD40 receptors, expressed on HRS cells [39]. This mechanism is critical in the pathogenesis of EBV-positive HL as one of the EBV latent proteins, LMP-1, acts as a constitutively activated CD40 receptor, and consequently prevents apoptosis [71].

### 2.6. Therapeutic Options

The standard treatment of EBV-positive malignancies is no different than that of EBV-negative malignancies, and consists of systemic chemotherapy and radiotherapy [72]. Novel approaches for the treatment of EBV infection is an area of intense research interest. Cellular therapies, immunotherapies, and antibody therapies are being developed and are collectively known as virus-targeted therapies. EBV-targeted therapies are designed to induce lytic replication, inhibit the expression of EBV-encoded oncogenes, induce loss of EBV episome, and promote the expression of toxic genes within EBV-positive tumor cells [55]. 

Induction of lytic gene expression results in the direct killing of virus-infected cells and increases the vulnerability of virus-infected cells to antivirals [56]. Arginine butyrate, histone deacetylase inhibitors, DNA methyltransferase inhibitors, radiation therapy, and B-cell receptor-blocking antibodies have been explored as lytic gene-inducing agents in animal models and small human trials [71,73,74]. The proteasomal inhibitor bortezomib and aspirin have also been shown to reactivate EBV lytic replication in vitro [75]. Interestingly, chemotherapeutic drugs, such as doxorubicin, gemcitabine, cisplatin, etoposide, 5-fluorouracil, and taxol, have shown EBV inducing abilities in various EBV-associated models [71,75]. However, the optimal inducing agents have yet to be determined due to limitations in the pharmacokinetics and pharmacodynamics of these agents in vivo [73]. 

Combination therapy is a promising advancement in the treatment of EBV-associated malignancies. This therapeutic approach is based on induction of EBV lytic gene expression, followed by exposure of the tumor cell to antiviral drugs [73]. Nucleoside analog antivirals, such as ganciclovir, acyclovir, or famciclovir are effective in suppressing viral replication during the lytic phase of EBV infection [73]. These antivirals require conversion to their active form by virus-encoded kinases, which are expressed only during the lytic state, and therefore, they are not effective in EBV-associated malignancies that maintain a latent state of replication [74]. Induction of EBV lytic gene expression allows for antivirals to be converted to the active form. Once activated, these antivirals prevent the synthesis of viral DNA, inhibit host cell DNA polymerase, and kill tumor cells [75]. One downside to lytic EBV induction is the increased risk of viral dissemination and transmission in the host. When these therapeutic approaches were tested in patients with nasopharyngeal carcinoma, the levels of viral DNA were increased, probably from apoptotic tumor cells [75]. Moreover, the risk of developing EBV-associated lymphomas has been reported to be higher following treatment with lytic inducing agents [75].

Expression of specific cellular contents, such as cytosine deaminase or thymidine kinase, also renders the cell susceptible to nucleoside analog antivirals [73]. Several studies have utilized EBV-dependent vectors to induce the expression of cellular toxins [73]. One example is an oriP-based vector. Currently, this approach is limited due to the lack of a reliable method to target the delivery of these vectors to only EBV-containing cells [73].

To date, there are no effective therapies that aim at latent EBV infection due to the fact that EBV utilizes host replication machinery while in latency, making it difficult to target EBV without affecting the host cell [75]. Therapies targeting EBV latent proteins and EBV episome are being investigated. Low dose hydroxyurea has been shown to eliminate EBV episome and decrease tumorigenic potential in BL cells in vitro and in severe combined immunodeficiency (SCID) mice [73]. Although its mechanism of action is not fully understood, hydroxyurea seems to alter the replication timing and chromatin organization of EBV’s origin of plasmid replication oriP [75]. Geminin, an inhibitor of mammalian replication initiation complex, also inhibits the replication from oriP [75]. However, the effectiveness of these approaches has yet to be evaluated in a human clinical trial.

Another strategy is to interfere with the functions of EBV latent proteins. Indeed, by inducing the expression of antisense RNA against EBV LMP-1 protein, a reduction in LMP-1 and Bcl-2 expression together with the inhibition of cell proliferation and stimulation of apoptosis were observed [73]. Cidofovir, an acyclic nucleoside phosphonate analog, is distinct from other antiviral drugs due to the fact that it can bypass the activation steps by viral-encoded kinases [75]. BL cells treated with cidofovir demonstrates a decrease in LMP-1 and EBNA-2 proteins, as well as a decrease in Bcl-2 and an increase in Bax, which result in inhibition of cell proliferation and induction of apoptosis [75]. In a clinical trial of 40 patients with nasopharyngeal carcinoma, DNAzyme specifically targeted at the LMP-1 mRNA lead to the downregulation of LMP-1 expression and an enhanced radiosensitivity together with short-term tumor regression [76].

Inhibition of signaling pathways mediated by latent proteins is another therapeutic area in development. Given the fundamental role of Syk signaling in B cell survival, several Syk inhibitors, such as fostamatinib (R788), entospletinib (GS-9973), cerdulatinib (PRT062070), and TAK-659, are being investigated in clinical trials [55]. In EBV-PTLD, inhibition of Syk led to increased apoptosis and decreased tumor proliferation [56]. Inhibitors of PI3K/AKT and its downstream target mammalian target of rapamycin (mTOR) are also in development [51,56]. A PI3K inhibitor, Ly294002, has been shown to induce apoptosis, inhibit cell growth, and enhance the effect of 5-fluorouracil in EBV-positive gastric cancer cell line [77]. Rapamycin, an mTOR inhibitor, was found to decrease tumor growth and metastasis in a mouse model of EBV-associated BL [78]. Dual inhibitors of PI3K and mTOR are in clinical development to treat cancer with activated PI3K. For example, BEZ235 [79,80] and SF1126 [81] are in phases 1 and 2 clinical trials. IPI-145, BYL719, BKM120, and BAY 80-6946 have entered into phase 3 clinical trials for the treatment of various cancers [82]. Idelalisib is the only approved dual inhibitor and is used in the treatment of chronic lymphocytic leukemia and two other lymphomas [82]. Although clinical trials that specifically evaluate the impact of PI3K inhibitors on EBV infection are lacking, pre-clinical data suggest that it may be useful to apply PI3K inhibitors in the treatment of EBV-associated malignancies.

Monoclonal antibodies and vaccines have been used to treat and prevent the development of many human pathologies, including some EBV-associated diseases. Rituximab, a monoclonal antibody against CD20 (a B cell specific protein), has been used in the treatment of PTLD [75]. However, rituximab targets all B cells expressing CD20 and does not selectively target EBV-containing cells [75]. Monoclonal antibodies against CD70, which is expressed by cells that are infected with latent II and III EBV, lead to inhibition of cell growth and are potential therapeutic options specifically for EBV infection that need further validation [75].

Vaccination with recombinant gp350 viral glycoprotein or CTL epitope-based peptide has been shown to generate viral immunity in animal models [73]. Clinical vaccine trials in healthy individuals demonstrated the development of neutralizing anti-EBV antibodies in vaccinated individuals [73]. Although the vaccine had little effect on the frequency of seroconversion, it did reduce the frequency of infectious mononucleosis [75]. The impact of vaccines on the development of EBV-associated malignancies is not defined. CTLs are important immune cells in the fight against EBV infection. EBV-positive donor CTLs have been utilized in the treatment of PTLDs and other solid tumors, with some clinical benefits in selected patients [72]. Levels of CTLs can be increased by generating immunity via CTL epitope-based peptide or CTLs can be isolated from the patient’s own lymphocytes, amplified ex vivo, and infused back to the patient [72,73]. Together, these novel approaches have shown promising results but will need further investigation in larger clinical trials to determine their efficacy and tolerability.

## 3. Human Herpesvirus-8

Human herpesvirus-8, also known as Kaposi sarcoma-associated herpesvirus, is another member of the herpesviridae family best known for its association with Kaposi sarcoma (KS). HHV-8 has also been implicated in two B-cell lymphoproliferative diseases, multicentric Castleman disease (MCD) and primary effusion lymphoma (PEL) [83]. Viral transmission occurs via salivary, blood, and sexual contact; mother-to-fetus transmission is rare [84].

The seroprevalence of HHV-8 worldwide is estimated to be between 5% and 20% [85]. North America, much of Europe, and Asia are geographic areas with low HHV-8 seroprevalence (<5%); the Mediterranean, Eastern Europe, Caribbean, and the Middle East have intermediate seroprevalence (5–20%); Africa and the Brazilian Amazon have the highest seroprevalence (>50%) [86]. In spite of its prevalence, few patients who are infected progress to the development of HHV-8-associated diseases [85,86]. The populations at greatest risk of developing HHV-8-associated diseases are those with compromised immune status.

### 3.1. HHV-8 Life Cycle

HHV-8 is a double-stranded DNA virus with a genome size of approximately 165 kb in length [87]. Like all herpesviruses, the life cycle of HHV-8 consists of two phases, latent and lytic replication. After primary infection, HHV-8 exists in a latent state as an episome and uses host cellular machinery for replication [83]. Similar to other herpesviruses, the primary reservoir for HHV-8 latency is the B lymphocyte. Endothelial cells are a second site of viral latency but are the primary infected cells in KS [84].

**Figure 2:**
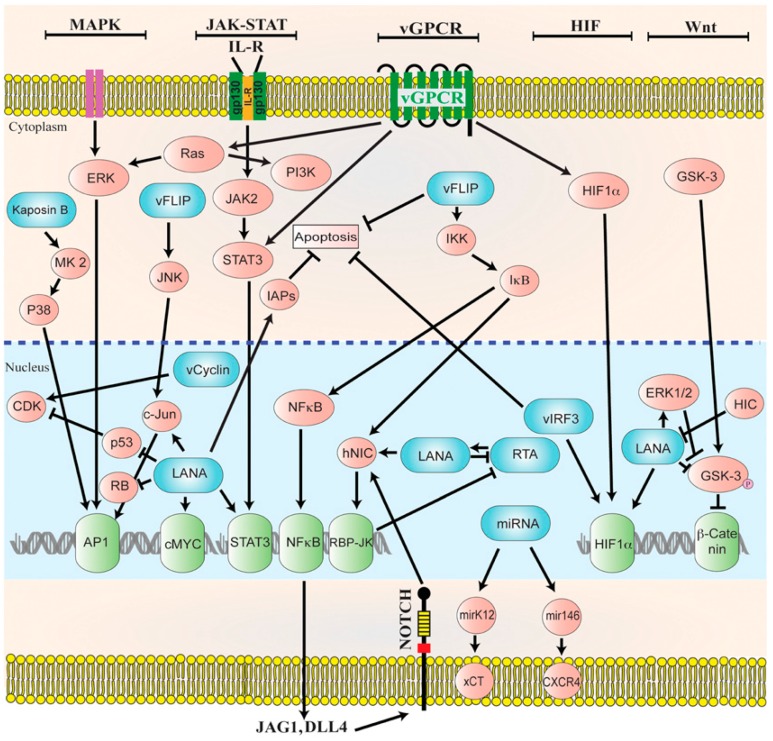
Diagrammatic representation of the cellular signaling pathways in maintaining latency. HHV-8 genome persists as a latent episome within the infected cells by expressing a limited number of viral genes during latency. For a successful establishment of latency, Human Herpesvirus-8 (HHV-8) manipulates and deregulates multiple viral and cellular signaling pathways. HHV-8 latent genes, including latency-associated nuclear antigen-1 (LANA), viral FLICE inhibitory protein (v-FLIP), microRNAs (miRNA), and viral Cyclin (v-Cyclin) activate and maintain various cytokine-mediated cell proliferation and tumorigenesis pathways, such as mitogen-activated protein kinase (MAPK), Janus kinase/signal transducer and activator of transcription (JAK/STAT), mitogen-activated protein kinase/extracellular-signal regulated kinase (MEK/ERK), phosphoinositide 3-kinase/protein kinase B/mammalian target of rapamycin (PI3K/AKT/mTOR), Notch, Wingless-related integration site (Wnt), cMyc, p53, retinoblastoma (RB), and nuclear factor-κB (NF-κB), to maintain latent infection. Reproduced with permission from Purushothaman, P., KSHV Genome Replication and Maintenance; published by *Front. Microbial.*, 2016.

In latency, HHV-8 can modulate host cellular signaling pathways that are involved in various cellular processes [83]. Latent proteins that are essential to the pathogenesis of HHV-8-associated malignancies include LANA, viral Cyclin (v-Cyclin), viral FLICE inhibitory protein (v-FLIP), kaposin, and many miRNAs (Figure 2) [88]. Latent infection of primary cells with HHV-8 creates a favorable microenvironment for tumor initiation and progression. However, unlike latently EBV-infected cells, latently HHV-8-infected cells do not become immortalized. Most spindle cell lines thata re isolated from KS lesions and grown in culture eventually lose the HHV-8 episomal genome after 5–10 divisions in the absence of reinfection, indicating that lytic replication is required for viral persistence [89].

While latency plays an important role in viral genome maintenance, lytic replication is essential for the preservation, transmission, and dissemination of the virus and the induction of an inflammatory microenvironment [90]. In the lytic state, HHV-8 produces linear DNA molecules that are then packaged into infectious virions. The switch between latent and lytic replication is regulated by the open reading frame (ORF) 50 gene encoding the replication and transcription activator (RTA) protein [85]. Various internal and external factors, such as inflammation, hypoxia, oxidative stress, epigenetic modifications, and even co-infection with HIV, HSV, HHV-6 and CMV, can trigger the virus to reenter lytic replication [90,91,92]. Amongst these, the immune status of the host appears to be the most important factor that controls viral reactivation [87]. 

### 3.2. HHV-8 Oncogenic Proteins

Amongst the latent viral proteins, LANA is the most consistently expressed protein in all of the HHV-8 infected tumor cells [87]. LANA is a key multifunctional protein in the maintenance of HHV-8 latency and oncogenesis [93]. LANA influences multiple cellular signaling pathways and cellular proteins, such as replication factors, transcription factors, and chromatin modifying enzymes, to maintain viral latency [93]. The suppression of cellular signaling pathways is essential for the evasion of the host immune surveillance. LANA directly inhibits signaling pathways, such as MAPK, JAK/STAT, ERK, PI3K/AKT, Notch, and Wnt [93]. Many of these pathways play a critical role during lytic reactivation, and must be deregulated in order to establish a successful latent infection. Furthermore, LANA suppresses lytic reactivation by silencing the transcriptional activity of RTA promoter [93]. It has also been shown that LANA promotes oncogenesis by enhancing cell survival and proliferation [87,91]. LANA-1 promotes cell cycle progression by inactivating the p53 and Rb tumor suppressor proteins [94,95]. Inhibition of p53 and Rb also impairs the apoptosis of HHV-8-infected cells [88,94].

Viral cyclin, a viral homologue of cyclin D, contributes to the maintenance of latency by forming a kinase complex with CDK6 and modulating cell cycle and cell proliferation [93]. Targets of vCyclin-CDK6 complex include Rb protein, histone H1, CDK inhibitors, and p27 [93]. The vCyclin-CDK6 complex is responsible for the phosphorylation and the inactivation of Rb protein, and thus promotes tumor formation [88]. The role of vCyclin in oncogenesis is also attributed to its suppression of p21 and p27 CDK inhibitors, and consequently stimulating cell cycle entry [95].

V-FLIP enhances cellular survival and proliferation during latency, mainly through its interaction with the NF-κB signaling pathway [93,96]. The mechanism by which v-FLIP activates NF-κB is not completely understood, although it is known that by constitutively activating NF-κB, v-FLIP upregulates transcription of anti-apoptotic genes, such as *BCL-2* [95]. V-FLIP inhibits apoptosis by interfering with Fas and TNF-mediated caspase activation and activates the NF-κB pathway by binding to the IκB kinase (IKK) complex [88]. One downstream effect of NF-κB activation is the increased expression of Notch ligand JAG1 [90]. Notch signaling contributes to key events in tumorigenesis, including sustained proliferation, angiogenesis, and EMT [90].

Kaposins are a set of latent proteins comprised of kaposin A, B, and C. Amongst them, kaposin B promotes tumor development by increasing cytokine expression [93]. Cytokines are important mediators of oncogenic processes, and a number of inflammatory cytokines are released in response to HHV-8 infection, creating a microenvironment that is conducive to tumor progression [94]. Significant increases in the levels of interleukin (IL) 6, TNF-α, MIP-1α, MIP-1β, and IL-8 have been observed in HHV-8-exposed, activated B cells as compared to unexposed cells during in vitro studies [97]. IL-6 and IL-10 serve as autocrine growth factors for MCD and PEL, respectively [87]. IL-8 binds to HHV-8-encoded viral G protein-coupled receptor (v-GPCR) to stimulate the production of angiogenic factors, as well as to induce expression of the lytic switch protein ORF50 RTA [97].

HHV-8 lytic genes include, *v-IL6*, *v-BCL2*, *v-MIP*, *v-GPCR*, and *viral IFN regulatory factor* (*v-IRF-1*) [94]. Lytic genes promote oncogenesis by altering DNA repair mechanisms, enhancing cellular survival, and favoring immune evasion. For example, v-GPCR promotes the activation of ROS, and thus induces oxidative DNA damage [95]; v-IL6 is a key factor for B-cell proliferation [94]; and, v-IRF-1 interferes with DNA damage response by inhibiting p53-mediated activation of the ATM protein kinase, a mediator of cell cycle arrest in response to DNA damage [95]. Moreover, v-GPCR and v-IRF-1 promote apoptosis resistance by activating the NF-κB pathway and inhibiting pro-apoptotic mediators, respectively [95]. V-GPCR also promotes angiogenesis by activating multiple signaling pathways that are deregulated during latent infection, such as MAPK, PI3K/AKT, and NF-κB, leading to the upregulation of angiogenic factors, like vascular endothelial growth factor (VEGF), IL-6, and platelet-derived growth factor (PDGF) [94,95].

### 3.3. HHV-8-Associated Tumors

Kaposi sarcoma (KS) is a low-grade mesenchymal tumor involving the lymphovascular system. It was originally described in 1872 by the Hungarian born dermatologist Moritz Kaposi as idiopathic multiple pigmented sarcoma of the skin [98]. For more than a century, KS was essentially unknown until the 1980s AIDS epidemic led to a surge in the incidence of KS by more than a thousand-fold in homosexual/bisexual men, intravenous drug users, and sexually promiscuous men and women. KS was observed to be more common among those who had acquired the human immunodeficiency virus (HIV) through sexual contact than parenterally prompting speculation of an infectious cause [99,100,101]. In 1994, HHV-8 was discovered in biopsy samples of KS [102], and has now been established as the etiologic agent in over 95% of all AIDS and non-HIV-associated KS.

MCD is a rare non-cancerous B-cell lymphoproliferative disorder characterized by lymph node hyperplasia [103]. On the other hand, PEL is a rare B-cell non-Hodgkin lymphoma (B-NHL) that is usually present as malignant effusions in body cavities without detectable solid tumor masses [88]. The majority of cases arise in patients with immunocompromised status, as in HIV infection or post-transplantation [88]. HHV-8 is present in all of the cases of PEL. In fact, the definitive diagnosis of PEL required the detection of HHV-8 in lymphoma cells, often via immunohistochemistry for latency-associated nuclear antigen-1 (LANA-1) [88].

### 3.4. Therapeutic Options

The current standard treatments for KS and PEL are combination antiretroviral therapy (ART) and chemotherapy. There are currently no standardized criteria to guide the selection of patients for chemotherapy. According to the WHO recommendations, patients that are diagnosed with mild to moderate KS can be treated with ART alone, while those with severe disease should be treated with ART in combination with systemic chemotherapy [104]. Local therapies, such as radiotherapy, topical therapy, and intralesional chemotherapy have been used in the pre-ART era for the management of localized and symptomatic disease [104]. However, local therapies can be more costly and less convenient for patients, which are some of the limitations to their use in the treatment of KS. Moreover, there are no specific recommendations regarding the use of local therapies for KS [104].

Owing to the number of chemotherapy-refractory cases, several molecular-targeted therapies are being explored in clinical and pre-clinical trials [88]. Since NF-κB activation is a key event in HHV-8 oncogenesis, various NF-κB inhibitors, such as cepharanthine [105], diethyldithiocarbamate [106], berberine [107], and heat-shock protein 90 [108], have been investigated and have shown promising preclinical results. Diallyl trisulfide (DAT) is a compound that is found in garlics that have antitumor effects. DAT have been shown to inhibit NF-κB signaling by blocking the IKK complex, and thereby reducing the viability of PEL cells [109]. Bortezomib, an NF-κB inhibitor, is currently in a phase 1 trial for patients with relapsed/refractory AIDS-associated KS (NCT01016730).

Several clinical studies have investigated novel therapeutic approaches for HHV-8-associated malignancies. Angiogenesis inhibitors are among the drugs being studied. Bevacizumab is a neutralizing antibody against VEGF that has been approved by the Food and Drug Administration (FDA) for the treatment of breast, lung, and colon cancer and is now being investigated for the treatment of KS. A phase 2 study of bevacizumab in HIV-negative and AIDS-associated KS found an overall response rate of 31% [110]. Patients who responded had been on a stable ART regimen with controlled HIV disease, and four of five responders had prior cytotoxic chemotherapy [110]. Another phase 2 study evaluating the efficacy of bevacizumab combined with liposomal doxorubicin, followed by bevacizumab monotherapy in patients with advanced KS is currently ongoing. Preliminary results show an overall response rate of 50% and 67% in patients with advanced KS not improved on antivirals and patients with all other advanced HIV-associated KS, respectively, after six cycles of bevacizumab combined with liposomal doxorubicin (NCT00923936). A phase 2 study in Mexico demonstrated that intralesional bevacizumab, combined with ART, resulted in a higher response rate when compared to ART alone in the treatment of KS of the airway [NCT01296815]. However, the findings in these studies are limited by the small sample size. Larger clinical trials are warranted to confirm the findings in these studies. Furthermore, the therapeutic impact of angiogenesis inhibitors as single agents is limited due to redundancy in the paracrine signaling [111]. However, they can potentially be effective adjunctive therapy to chemotherapy. 

Many other drug classes are being investigated for the treatment of HHV-8-associated diseases, including IL-2, immunomodulating agents, mTOR inhibitors, matrix metalloproteinase (MMP) inhibitors, MAPK kinase inhibitors, and receptor tyrosine kinase (RTK) inhibitors. PEL expresses cellular markers, such as CD30, that are potential therapeutic targets. Brentuximab vedotin, an antibody-drug conjugate that selectively targets CD30, has been demonstrated to prolong survival in the PEL xenograft mouse model [112]. RTK inhibitors, such as sorafenib and imatinib, have some activities against KS. Sorafenib has modest anti-KS activity, but was poorly tolerated when used in combination with ritonavir, a CYP3A4 inhibitor [110]. Imatinib, a c-kit/PDGF-receptor inhibitor, also has modest anti-KS activity, with thirty percent of patients having long-term clinical benefits, and is well-tolerated [113]. One paper reported the complete regression of KS after treatment with imatinib in a patient with highly chemoresistant disease [114], suggesting that imatinib may be an alternative option for patients who have failed chemotherapy.

A number of antivirals have previously been reported to block HHV-8 DNA synthesis [89]. Amongst these agents, ganciclovir/valganciclovir is the only one shown to inhibit HHV-8 replication in a placebo-controlled trial of 26 HHV-8 infected men [115]. In a case series of 3 patients with MCD, administration of ganciclovir resulted in symptom improvement and reduction in HHV-8 plasma levels [115]. Ganciclovir/valganciclovir have also been shown to prevent the development of KS in small, randomized controlled trials [116,117]. However, a more recent study of oral valganciclovir in the treatment of five HIV-seronegative patients with KS did not find an effect on tumor growth or HHV-8 gene expression [118]. The impact of other antivirals on HHV-8 infection has also been explored, although the evidence for these agents is weaker. Few small studies have demonstrated an association between foscarnet and reduced KS incidence [117,119].

The benefit of antivirals for the treatment, as opposed to prevention, of KS and other HHV-8-associated diseases is less clear [89]. Foscarnet and cidofovir have been suggested to improve KS and PEL treatment outcomes in case reports [120,121,122]. However, the largest pilot study of cidofovir in seven patients with HHV-8-associated KS found no effect on disease progression, latent or early lytic gene expression, or HHV-8 viral load [123]. Valacyclovir and famciclovir were associated with reductions in HHV-8 shedding frequency [124]. On the other hand, in vitro and in vivo studies have shown no efficacy of acyclovir against HHV-8 replication or HHV-8-related diseases [125,126]. To date, there are no large clinical trials or definitive data to prove the benefits of these antivirals against HHV-8. At this time, there is insufficient evidence to determine who might benefit most from the prophylactic use of antivirals against HHV-8-associated diseases [89].

## 4. Merkel Cell Polyomavirus

Merkel cell polyomavirus (MCPyV) is a non-enveloped circular double-stranded DNA virus of genera *Orthopolyomavirus* and family *Polyomavirida*. Simian virus 40 (SV40) and the human polyomaviruses JC and BK belong to the same family [127]. MCPyV is the only polyomavirus that is known to be oncogenic [128,129,130,131,132,133] and is found in 80–97% of Merkel cell carcinoma (MCC) [127,134,135]. MCC is an extremely rare and aggressive cutaneous cancer with an incidence and mortality rates of 0.79 and 0.43 per 100,000, respectively [136]. Incidence of MCPyV in MCC tumor is lower in countries with high UV indexes, such as Australia [137].

MCPyV is also found on 40–80% of healthy skin [138,139] and is present less frequently in the respiratory tract [139,140,141,142,143], saliva [144,145], lymphoid tissues [145,146,147], urine [148,149,150,151], and the gastrointestinal tract [128,152]. RAF-inhibitor associated cutaneous squamous cell carcinoma and verruca vulgaris are also frequently MCPyV-positive, but a causative role for MCPyV has not yet been established [153,154]. 

The mode of MCPyV transmission is unknown, although direct cutaneous contact is likely for several reasons: infected individuals cutaneously shed the virus in assembled virions [155], viral loads are higher in the skin than other locations, and MCPyV is also present on environmental surfaces [128,139,144,155,156]. Respiratory [140,143,157] and gastrointestinal transmissions [144,148,152] are also plausible. MCPyV infection occurs early in life. By ages 1–5 years, 20–40% of children are seropositive for MCPyV capsid antibodies [133], and 80% of the population is seropositive by age 50 [157,158]. Patients with MCPyV-positive MCC have much higher viral levels and antibody titers than MCPyV-positive patients without MCC [157]. 

### 4.1. MCPyV Genome

**Figure 3:**
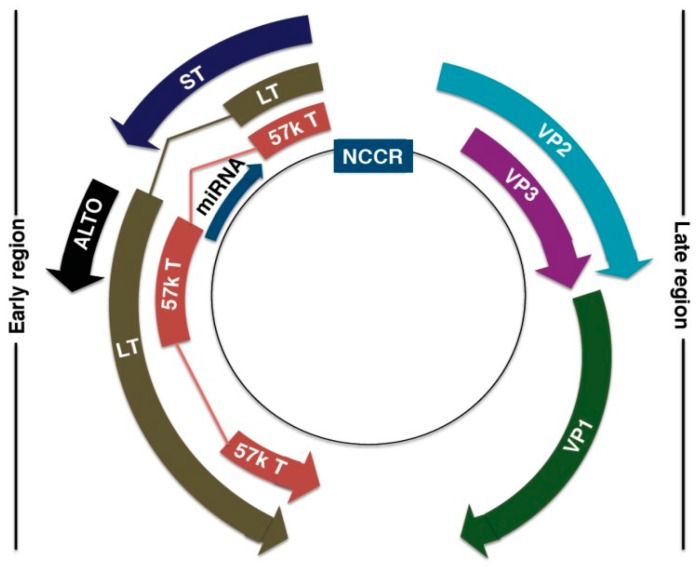
Merkel Cell Polyomavirus (MCPyV) genome organization. Non-coding control region (NCCR): bipartite origin of replication. Early gene region: Large T antigen (LT), small T antigen (ST), 57kT antigen (57kT), alternative T antigen open reading frame (ALTO), microRNA (miRNA). Late gene region: capsid proteins (VP1-3). Reproduced with permission from Stakaitytė, G., Merkel Cell Polyomavirus: Molecular Insights into the Most Recently Discovered Human Tumour Virus; published by *Cancers*, 2014.

The MCPyV DNA genome is ~5.4 kb, and is divided into three regions: the non-coding regulatory region (NCRR), early coding region, and late coding region [127,128,159] (Figure 3). The NCRR contains the origin of replication and transcriptional regulatory elements [127,159]. Early-coded proteins are expressed upon cellular infection before DNA replication and may assist in DNA replication, while late-coded proteins are produced after DNA replication [131]. Early-coded proteins include large T (LT) antigen, small T (ST) antigen, 57kT antigen, and the alternative LT open reading frame (ALTO) [159,160,161]. LT, ST, and 57kT antigens share a conserved N-terminus alternative, with alternative splicing downstream of the first exon [131]. LT and ST antigens are the oncoproteins that drive MCC tumorigenesis [159,162]. The role of 57kT antigen is largely unknown, but its homologous counterpart in SV40 promotes host cell proliferation [163,164]. ALTO allows for protein diversity within a limited genome. Although not essential for MCPyV replication and tumorigenesis, ALTO is mutated in many cancer tissues [161].

Late-coded proteins include major capsid protein VP1 and minor capsid proteins VP2 and VP3 [133]. VP1 is thought to mediate cell attachment and entry through interaction with glycosaminoglycan, such as heparan sulfate, and ganglioside on the host cell membrane [138,165]. Production of highly immunogenic VP1 decreases in MCC development, lessening the host’s immune response to MCPyV [133,166,167,168]. VP2 contributes to infectivity, as indicated by a reduced infectivity in VP2-knockout mice [169]. However, VP2 appears less essential to cell entry, as it does not directly affect trafficking, viral DNA packaging, or binding to cellular receptors [165,169]. VP3 is not detected in native MCPyV or MCPyV-infected cell lines [169].

**Figure 4:**
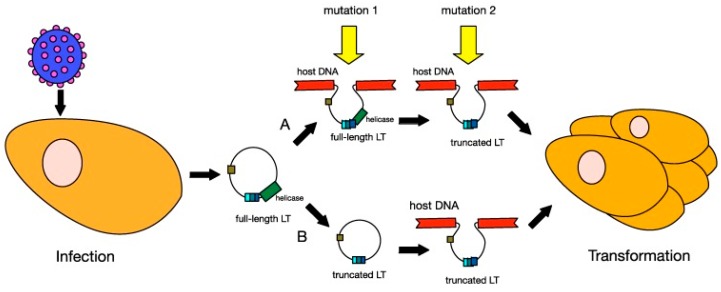
Models of MCPyV-induced Merkel cell carcinoma (MCC) tumourigenesis. MCPyV infection is thought to occur early in childhood of most people. Before tumourigenesis can occur, loss immunosurveillance must lead to proliferation of the virus. At least two mutations are needed before MCPyV can transform cells. In model A, the first mutation is thought to be the integration of the full-length viral genome into host DNA, while the second mutation is the truncation of LT. In model B, truncation of LT is thought to occur before integration. Either way, these changes in the virus lead to cellular transformation and tumour proliferation. Reproduced with permission from Stakaitytė, G., Merkel Cell Polyomavirus: Molecular Insights into the Most Recently Discovered Human Tumour Virus; published by *Cancers*, 2014.

In MCC, MCPyV DNA clonally integrates into the cellular DNA as a result of mutation [128,135,170,171] (Figure 4). Distant metastatic MCC cells have the same clonal copies of MCPyV DNA as primary tumor cells, suggesting that MCPyV clonal integration drives oncogenesis [133,150,172]. Clonal integration does not occur in MCPyV-infected individuals who do not have MCC [128,150]. MCPyV clonal integration and protein expression were found to occur in human dermal fibroblasts, but not epidermal cells [173], supporting a MCC dermal tumor origin hypothesis. Furthermore, MCC is typically located in the dermis and subcutis with rare extension to the epidermis [174]. 

### 4.2. MCPyV Oncogenic Factors

In addition to the mutation causing clonal integration, a second mutation is necessary for MCC development. Perhaps the requirement of these two mutations is why MCC is exceedingly rare, despite cutaneous MCPyV infection being common [134]. The second mutation may arise from UV radiation [175] and results in the truncated form of the LT antigen [129] (Figure 4). The mutation occurs in exon 2 as stop codon mutations or as pre-mature integration break points [146,150,176,177,178]. As a result, the LT antigen is truncated at the C-terminus [160,176]. The C-terminus contains the origin-binding domain, helicase, and replicase [173,179]. The loss of this region eliminates viral replicative abilities, and MCPyV DNA survival must remain clonally integrated for survival [173,179]. Likewise, the transformed cells become dependent on LT and ST antigen expression for proliferation and survival [162]. 

The N-terminus, including the J domain, and LxCxE motif are conserved during LT antigen truncation. The N-terminus and J domain contain the Cr1 epitope (LXXLL) and DnaJ (HPDKGG) domain, which binds hsc-70 during replication [128,165]. The LxCxE motif binds and inactivates the tumor suppressing Rb protein [160,176]. Rb is then unable to bind to E2F and prevent entry into the S-phase of the cell cycle [165]. The DnaJ domain further prevents entry into the S phase by facilitating E2F release in an ATP dependent fashion [180]. LT antigen also contributes to oncogenesis by inhibiting apoptosis, stimulating telomerase activity, and inducing angiogenesis [133]. In essence, the MCPyV DNA clonally integrates into cellular DNA via truncation of the C-terminus, but MCC cells evade the immune system through N-terminus activity. 

Unlike LT antigen, ST antigen does not require mutations to have an oncogenic effect [48]. ST antigen induces MCC oncogenesis by promoting the cap-dependent translation downstream of the mTOR phosphorylation pathway [132,181]. Cap-dependent translation is initiated by the mRNA-binding eukaryotic initiation factor 4E (eIF4E) complex [181]. 4E binding proteins (4E-BP1) regulate cap-dependent translation by preventing eIF4E formation [181,182]. The LT stabilization domain (LSD) of ST antigen inactivates 4E-BP1 by hyperphosphorylation, allowing for unregulated cap-dependent translation [181,182]. LSD accomplishes this by binding cell division cycle protein 20 (Cdc20) and possibly cdc20 homolog 1 (Cdh1) E3 ligase adapters [181]. These binding events activate the cyclin-dependent kinase 1/cyclin B1 (CDK/CYCB1), which directly hyperphosphorylates the 4E-BP1 [181]. LSD also prevents the proteasomal degradation of the LT antigen via the SCFFBw7 E3 ligase [183]. 

ST antigen may contribute to oncogeneis in several other ways, including aerobic glycolysis promotion [184] and ST antigen-mediated c-Jun phosphorylation [184], a process that is known to contribute to multiple cutaneous malignancies [185,186]. Protein phosphatase 2A targeting does not appear to be integral to MCPyV ST antigen-induced oncogenesis, unlike nonhuman polyomavirus oncogenesis [181,187].

LT antigen was originally thought to be more oncogenic than ST antigen, but many now believe that ST antigen is more critical for MCC tumorigenesis for multiple reasons. Unlike LT antigen, ST antigen alone was sufficient to induce transformation in rodent fibroblasts [181]. ST antigen is also found more commonly in MCPyV MCC than LT antigen, and knockout of ST antigen alone was almost as effective at impeding MCPyV-infected cellular survival as pan T antigen knockout [181]. However, the small difference in cellular survival between ST antigen knockout and pan T antigen knockout suggests that both ST and LT antigen oncogenic activity are required for optimal tumorigenesis [181]. 

The precise role of the p53 tumor suppressor pathway in MCPyV-positive MCC is unknown. Shuda et al. showed that ST antigen alone could cause neoplastic transformation in p53-null mice, but not p53-positive mice [132]. p53 inactivity may therefore be important for MCPyV-positive MCC oncogenesis. However, p53 mutations are common only in MCPyV-negative MCC [188] so p53 pathway inactivation in MCPyV-positive MCC is likely to occur upstream or downstream of p53 production [132]. 

Full-length LT antigen with intact C-terminus induces p53 activation in multiple cell lines, causing cell cycle arrest and decreased proliferation [189]. Truncated LT antigen does not activate the p53 pathway, and thus avoids tumor suppression [189]. Another study suggested that full length LT antigen indirectly inhibits the p53 pathway [190], making LT antigen’s interaction with the p53 pathway unclear. 

### 4.3. Therapeutic Options

NCCN MCC management guidelines are the same for MCPyV-positive and MCPyV-negative tumors (NCCN) with viral-positive tumors having a better prognosis [191]. MCPyV-positive MCC prognosis may improve as many of the current research is focused on MCPyV-specific therapy. Survivin is an oncoprotein crucial for the survival of MCPyV-positive MCC cells [192], and a potential therapeutic target. YM155, a survivin inhibitor, caused selective programmed cell death in MCPyV-positive MCC tumor cells in mouse models [192,193]. MCPyV vaccines have been successful in murine models at inducing cell-mediated immunity and increasing survival. Successful murine vaccines include an LT antigen-targeting DNA vaccine [194] and ST antigen-targeting vaccine [195]. Additional MCPyV epitopes have been identified as potential vaccine targets [196]. To date, vaccines have not yet been studied for human use. The recently identified association of c-Jun hyperactivation with MCPyV ST antigen may be another potential therapeutic target [184]. 

Although immunosuppression increases the risk of MCC, most MCPyV-positive MCC patients are immunocompetent. These patients are still unable to clear MCC tumor cells, despite the presence of immunogenic foreign MCPyV capsid and oncoproteins. MCPyV may elude the immune system through the downregulation of MHC-1 [197], Toll-like receptor 9 (TLR9) [198], and genes associated with NF-κB pathways [199]. Immunotherapy options are currently being employed and investigated. 

The FDA has recently approved avelumab, a fully human anti-PD-L1 monoclonal antibody, to treat stage IV MCC [200]. Extended results of a phase II clinical trial revealed treatment response in 33% of patients (11.4% complete, 21.6% partial) with a median response time of six weeks and median overall survival of 11.3 months [200,201]. Pembrolizumab and nivolumab are additional anti-PD-L1 antibodies that have shown promise in treating stage IV MCC in clinical trials and case reports, respectively [202,203,204]. No relationship was found between MCPyV status and treatment response with avelumab or pembrolizumab [200,202]. 

## 5. Human Papillomavirus

Human papillomavirus (HPV) is a double-stranded DNA virus of the *papillomavirus* family [205]. More than 200 different HPV types exist, which can be classified into five genera: α, β, γ, ν, and μ [206]. HPV is the most commonly sexually transmitted disease [205,206], and infects epithelial cells via skin-to-skin or mucosa-to-mucosa contact [206]. The lifetime risk of sexually-transmitted HPV infection is 50% [207]. HPV tissue tropism varies by genera, with α-papillomaviruses infecting mucosa and β- and γ-papillomaviruses infecting skin [208]. HPV types are divided into low-risk (LR) and high-risk (HR) types, with HR carrying an increased risk of cancer development. All of the HR HPV types are of the α genera [206]. The most common HR types are HPV 16, 18, 31, 33, 52, and 58, and the most common LR types are HPV 6, 11, and 53 [206]. HPV 16 and 18 are the most common types worldwide, and the primary types linked to carcinogenesis [206]. Malignant manifestations of HPV include cervical, penile, vulvar, vaginal, anal, and oropharyngeal carcinoma [209]. Benign cutaneous manifestations of HPV include common warts (verruca vulgaris), plantar warts, plane warts, anogenital warts, and epidermodysplasia verruciformis. Benign mucosal manifestations of HPV include oral warts, condylomata, focal epithelial hyperplasia (Heck’s disease), nasal and conjunctival papillomas, laryngeal papillomas, and cervical lesions [210,211]. 

HPV accounts for more than 50% of infectious cancers in women and 5% in men [212]. Most HPV infections, however, are cleared by the host’s immune system within 1–2 years [213,214]. α-HPV DNA is found in more than 99% of cervical cancer, 85% of anal cancers, and 50% of penile, vulvar, and vaginal cancers [205]. Multiple preventative HPV vaccines have been developed for females and males with the hopes of preventing HPV-driven cancers. These vaccines have proved efficacious in decreasing the prevalence of persistent HPV infection. Further study will demonstrate the effects of HPV vaccination on the development of cancer [214,215]. 

### 5.1. Structure and Genome

HPV is a 55–60 nm non-enveloped circular double-stranded DNA icosahedral virus of ~8 kb pairs [206,207,208]. The genome is composed of an early-coded region (genes E1–E3, E4–E7), late-coded region (genes L1 and L2), and non-coding control region [216]. Genes L1, L2, E1, and E2 are well-conserved across papillomaviruses [208]. A distinct HPV type is designated when the DNA sequence of the L1 ORF differs from that of other types by at least 10% [217,218]. 360 molecules of the 72 pentameric major capsid protein L1 form the external viral capsid and mediate cellular binding. L2, the minor capsid protein, forms the inner surface of the viral capsid with the N-terminus extending to the capsid surface. L2 contributes to infectivity, viral trafficking, encapsulation of viral DNA, virion release, and the suppression of maturation of Langerhans cells [216,219,220]. E1 and E2 play roles in viral replication: E1 encodes a viral-specific helicase necessary for DNA replication and amplification, while E2 participates in viral transcription, replication, and genome partitioning [221]. E3 does not exist, which is likely due to an error in genome sequencing [221]. E4–E7 carry a greater amount of genetic diversity than the core genes, which accounts for the differences in infectivity amongst different HPV types [221]. E4 helps evade host epithelial defenses [221]. E6 and E7 are the primary oncoproteins, with E5 also contributing to cancer development. The primary differences between HR and LR HPV is due to genetic variations in E6 and E7 [205,222,223]. E6 and E7 inhibit tumor suppressor pathways. E6 utilizes the ubiquitination-mediated pathway to target and degrade p53 [205,222,223], while E7 inhibits the retinoblastoma protein and activates the oncogenic transcription pathways through interaction with histone deacetylases [206,211]. S-phase protein expression is subsequently increased, which triggers re-entry into the cell cycle and initiation of DNA synthesis [211]. E6 and E7 both alter cytokine expression in addition to activating PI3K/AKT, Wnt/β-catenin, and Notch signaling pathways [206,224]. Through all of these actions, E6 and E7 increase cellular proliferation, decrease apoptosis, alter cell cycle regulation and telomere maintenance, and induce DNA damage and genomic instability [206].

### 5.2. Pathogenesis and Carcinogenesis

The carcinogenic role of HPV has been studied to a great extent in cervical cancer, and it is assumed that HPV has a similar, albeit not identical, role in other HPV-associated cancers. The following description applies to HPV in cervical cancer. HPV infects basal cervical epithelial cells following mucosal microtrauma occurring during sexual intercourse [225]. HPV 16, the most common cause of cervical cancer, initiates cellular entry via L1 interaction with heparin sulfate proteoglycans (HSPGs). This facilitates the transfer of HPV to a receptor complex prior to cellular internalization of the virus [219]. L1 is then uncoated from the virus in the endosomal trafficking system, which exposes L2 associated with HPV DNA. The L2/DNA complex travels through the Golgi network and endoplasmic reticulum, before arriving at the nucleus where DNA replication can occur [219]. 

HPV can only successfully replicate in dividing epithelial cells that undergo maturation, senescence, and cellular death [226]. Viral replicative activities occur in all of the layers of the epithelium, but virions are only assembled and released in the most superficial cell layer. The life cycle of HPV spans 2–3 weeks, corresponding to the time that it takes basal cervical epithelial cells to mature and progress to the superficial layer [227]. If HPV infection persists, the number of viral episomes per cell increases, and the likelihood of viral genome integration into the host genome increases. Genome integration encourages genomic instability and tumor formation [228,229]. HPV DNA is integrated in the majority of cervical cancers, and many believe this to be the key transforming event [230,231]; however, the majority of HPV-associated head and neck cancers do not have integrated HPV DNA [232]. Following genome integration, the HPV E2 ORF is disrupted, and E2 is no longer expressed. E2 normally regulates transcription of E6 and E7. Loss of E2 increases levels of oncoproteins E6 and E7, encouraging cell survival [225]. As the cells continue to divide, genomic instability increases and chromosomal abnormalities, such as methylation of viral and cellular genomes, continue to accrue and increase the likelihood of tumor development [226]. 

### 5.3. Cervical Cancer 

Cervical cancer is the fourth most common cancer in women, with 530,000 new cases and 270,000 deaths occurring annually [209]. The majority of cases occur in the developing world. The United States will have an estimated 12,820 new cases in 2017, with 84% of cases occurring at age 35 or older, and with a median age of diagnosis of 49 years. Overall survival in the U.S. at five years is 67.1%, with a projected 4210 deaths for 2017 [233]. 

In addition to HPV infection, risks factors for cervical cancer include early onset of sexual activity, multiple sexual partners, smoking, low socioeconomic status, and history of other sexually-transmitted infections [234]. HPV infection typically occurs in young women between 18 and 30 years of age, whereas cervical cancer is uncommon prior to age 35. This suggests that carcinogenesis takes years to develop [226]. The most common types of cervical cancer are squamous cell carcinoma (69% of cases) and adenocarcinoma (25% of cases) [235]. HPV 16 is found in 59.3% of squamous cell carcinoma and 36.3% of adenocarcinoma of the cervix [236]. HPV 18 is the second most common subtype in cervical cancer, and is more common in adenocarcinoma (36.8%) than squamous cell carcinoma of the cervix (13.2%) [236,237]. About 10% of women with normal cervical cytology are infected with HPV; many will never develop abnormal cytology, as most cervical HPV infections are cleared by the host in 6 to 18 months [226]. 

The transformation zone between the columnar epithelium of the endocervix and squamous epithelium of the ectocervix is the most common site of persistent HPV infection, cervical intraepithelial neoplasia, and cervical cancer [225]. The development of cervical cancer follows a progressive course: HPV infection of the transformation zone, failure to clear the virus, development of cervical intraepithelial neoplasia (CIN), and the invasion of epithelial basement membrane [238]. 

CIN has three different levels of severity: CIN 1, CIN 2, and CIN 3. CIN 1 is an insensitive sign of HPV infection, typically regresses, and usually does not progress to cancer [226,239]. CIN 2 is considered to be premalignant but can be caused by carcinogenic and noncarcinogenic HPV types [240]. CIN 3 represents severe dysplasia, or dyskaryosis, or carcinoma in situ. CIN 3 is caused by the carcinogenic HPV types and has the same risk factor profile as cervical cancer. CIN 3 is also more likely than other grades of neoplasia to exhibit HPV genome integration [240]. HPV 16 is found in roughly half of high-grade CIN, followed by types 18, 31, 33, and 58 [241].

To identify cervical cancer and its precursor lesions, the United States Preventative Services Task Force (USPSTF) recommends cervical cancer screening for all women, beginning at age 21. From age 21 to 30 years, women should undergo Pap smear cytology screening every three years. From age 30 to 65 years, women can continue Pap smears every three years or opt for Pap smears with HPV testing every five years. HPV testing may also be used to guide management when cytology reveals atypical squamous cells of undetermined significance (ASC-US). USPSTF guidelines may be modified and expanded for immunosuppressed patients or patients with increased risk factors [242]. 

### 5.4. Oropharyngeal Cancer

Oropharyngeal squamous cell carcinoma (OPSCC) rates have been decreasing in the United States due to lower smoking rates, but the incidence of HPV-positive OPSCC is rising by 5% annually [243,244]. Rising incidence is presumed to be from a higher proportion of the population engaging in oral sex, earlier onset of sexual activity, and an increased number of sexual partners [205,245]. HPV-positive OPSCC is associated with Caucasian race, male sex, age less than 60 years, and infrequent p53 gene mutations [213,243,244,246]. Only 39% of HPV-positive OPSCC contain clonally integrated HPV genomes, with integration most frequently occurring within common fragile sites (CFS regions): FRAXC (Xq22.1), FRA3C (3q27), FRA9D (9q22.1), and FRA17B (17q23.1) [232]. HPV 16 is the most commonly found type in HPV-positive oropharyngeal (90%) and oral (96%) cancers [247]. HPV-positive tumors exhibit less differentiation and a lower degree of aneuploidy than HPV-negative tumors [248], while also demonstrating better clinical outcomes and response to treatment [205,249]. Clinical trials are investigating if less intense treatment regimens can be used for HPV-positive tumors to achieve equivalent clearance and survival, while also decreasing treatment-associated morbidity [205,250]. Some trials have already demonstrated good oncologic control via more conservative surgical and radiation treatment regimens, allowing for an improved quality of life by decreasing dysphagia and lowering esophageal/mucosal adverse events [250,251]. 

### 5.5. Penile Cancer

Penile cancer is rare, comprising only 0.24% of neoplasms in the United States [252]. Approximately 47–80% of penile squamous cell carcinoma (SCC) is positive for HPV [253,254]. HPV 16 and 18 are present in 28.3% and 6.3% of penile SCC, respectively [255]. About 20% of men are positive for penile HPV, but most infections resolve within one year without development of cancer [256]. 

Progression to penile SCC may occur via an intermediate dysplastic premalignant lesion, penile intraepithelial neoplasia (PeIN), which is positive for HPV 60–100% of the time [255]. PeIN is categorized as erythroplasia of Queyrat of the glans and foreskin, Bowen disease of the keratinized penile shaft, or bowenoid papulosis [255]. HPV appears to have a stronger link with basaloid penile SCC (75% cases) and warty penile SCC (47.4% cases), than with the more keratinized subtypes of verrucous, papillary, and sarcomatoid penile SCC [255]. 

### 5.6. Anal Cancer

Anal SCC has an incidence of less than 2/100,000 worldwide [257], but the incidence is increasing [258]. HPV is present within 80–90% of anal cancers [259]. Anal intercourse is a risk factor for HPV infection, but is not required for transmission [260]. Additional risk factors for HPV transmission include oral sex with a male, lifetime number of sexual partners, and history of perianal and/or vulvar condyloma. Risk factors for persistence of anal HPV infection and/or anal SCC include douching, long-term tobacco use, anal intercourse, and immunosuppression [261]. Chronic irritation of the anal tract in the form of fissures or fistulas is an HPV-independent risk factor for anal SCC [261]. Anal epithelial neoplasia (AIN) is believed be a precursor of invasive anal SCC, similar to CIN 3 for cervical cancer [261].

### 5.7. Vulvar & Vaginal Cancer

Vulvar and vaginal SCC are uncommon, making up 5.6% and 4.7% of female genital tract cancers, respectively [262]. The mean age at diagnosis of vulvar and vaginal SCC is in the fifth or sixth decade of life [263,264]. HPV is detected in 40–70% of vulvar and vaginal SCC, as well as 85–90% of premalignant vulvar intraepithelial neoplasia grades 2 and 3 (VIN 2/3) and vaginal intraepithelial neoplasia grades 2 and 3 (VaIN 2/3) [265]. Unlike cervical cancer, no routine screening is performed for vulvar or vaginal SCC [263]. Vulvar SCC that is associated with HPV infection has an earlier age of onset and is correlated with lesser stage and negative lymph node metastasis as compared to HPV-negative vulvar SCC [266]. 

### 5.8. Preventative Vaccines 

Several preventative HPV vaccines are available. Gardasil is a quadrivalent vaccine against HPV types 6, 11, 16, and 18, and Cervarix is a bivalent vaccine against HPV types 16 and 18. Gardasil and Cervarix were licensed for use in the United States in 2006 and 2009, respectively [267]. Gardasil 9, a 9-valent HPV vaccine produced by Saccharomyces cerevisiae, which covers types 6, 11, 16, 18, 31, 33, 45, 52, and 58, was licensed by the FDA in 2014, and is now the only HPV that is produced for use in the United States [268]. CDC guidelines recommend that males and females be vaccinated between the ages of 9 and 26. Those whose vaccination schedule is initiated before age 15 are recommended to receive two doses, which are administered 6–12 months apart. Patients who are immunocompromised or whose vaccination occurs after age 15 should receive three doses [269]. 

The 9-valent vaccine has potential for great impact, as the five additional HPV types not covered by the 4-valent vaccine are responsible for 10% of invasive HPV-associated cancers (14% for females; 4% for males), 15% of cervical cancers, and 25% of CIN 2 [267]. All of the preventative vaccines are based on synthetic recombinant L1 major capsid protein, arranged in icosahedral virus-like particles [211,270]. A downfall of L1-based vaccines is that they induce HPV type-specific immune responses [220]. L2 minor capsid protein vaccines are being explored as a potential for broad-spectrum coverage due to the homology of L2 across HPV types. Animal models have shown promise for efficacious L2-based vaccines. In humans, an HPV fusion vaccine containing L2 appeared to have broad coverage, but limited efficacy due to low antibody titer response [271,272]. Multiple other L2 and L1/L2 vaccines are currently under study [220].

### 5.9. Therapeutic Immunotherapy 

Multiple clinical trials have been performed and are currently underway regarding therapeutic vaccines for HPV-induced cancers and premalignant HPV-driven changes such as CIN and AIN [211,273,274]. Preventative HPV vaccines develop humoral immunity, whereas the aim of therapeutic vaccines is to induce an antigen-specific cellular-mediated immunity. Vaccine-induced CD8^+^ cytotoxic T cells and CD4^+^ helper T cells target epithelial cells containing viral oncoproteins E6 and E7 [211,273,275,276]. 

Viral-based, peptide/protein-based, nucleotide-based, and whole cell-based vaccines are being explored [273]. Monoclonal antibody therapies, such as nivolumab, are also being studied as adjuncts to therapeutic vaccines (NCT02426892). Although preclinical and clinical trials have shown the success of HPV therapeutic vaccines in reducing tumor burden and resolving premalignant lesions, further investigation is required before a vaccine will be available as a treatment option.

## 6. Hepatitis B Virus

Hepatitis B virus is a member of the genus *Orthohepadnavirus* and family *Hepadnaviridae*. HBV can cause acute and chronic viral hepatitis and long-term complications, ranging from fibrosis to cancer. The likelihood of progression from acute to chronic infection depends on the age at the time of infection [277,278]. Most immunocompetent adults successfully clear the acute infection and less than 5% of adults progress to chronic hepatitis. Progression to chronic infection is more likely in patients infected during perinatal and childhood period, with approximately 90% and 20% of cases, respectively, persisting into the chronic phase [279].

An estimated 2 billion people are infected with HBV worldwide, and more than 350 million of those are chronic carriers [277,280,281]. Approximately 4.5 million new HBV infections are reported each year globally [277]. Highly endemic areas, such as Central Asia, Southeast Asia, Sub-Saharan Africa, and the Amazon basin, have chronic carrier rates over 8% [277,280]. The United States, Northern Europe, Australia, and parts of South America are considered areas of low endemicity with a carrier rate of less than 2% [277,278,280]. HBV causes significant morbidity and mortality accounting for more than 600,000 deaths each year due to acute disease or chronic sequelae [277,278,280].

Viral transmission primarily occurs through percutaneous or mucosal contact with infected body fluids, like saliva, tears, semen, vaginal secretions, and blood. Routes of transmission include, sexual intercourse, parenteral contact, and perinatal [277,278,280]. HBV infection acquired through blood transfusion was a major problem in the past, but its incidence has significantly declined after strategies for blood donor screening for hepatitis B surface antigen (HBsAg) and hepatitis B core antibody (anti-HBc) were implemented in 1971 and 1987, respectively, in the United States [282].

Hepatitis B virus is a well-established risk factor for the development of hepatocellular carcinoma (HCC). The risk of developing HCC increases by 40% in patients with chronic HBV infection [283]. HBV accounts for 20% of HCC cases in the U.S., Europe, and Japan, and 60% of cases in Asia and Africa [284,285]. Development of HCC usually occurs within 10–30 years after the initial HBV infection due to an accumulation of mutations [286,287]. The oncogenic mechanisms in HBV-related HCC involve both direct and indirect pathways via direct oncogenic activities of specific viral proteins and chronic inflammation, respectively [284]. HBV has also been associated with several other malignancies, such as B-NHL [288] and nasopharyngeal carcinoma (NPC) [289], although the exact pathogenesis in these cancers remains unclear.

### 6.1. HBV Genome

The HBV genome is a 3.2 kb partially double-stranded relaxed circular DNA comprised of four open reading frames: *surface* (*preS1*, *preS2*, *S*), *core* (*precore*, *core*), *polymerase*, and ‘*x*’ genes [284,290]. HBV replicates via a DNA polymerase with reverse transcriptase activity similar to retroviruses [284]. The replication process of HBV is inherently error prone since it occurs via an RNA intermediate and lacks proofreading activity, which results in mutations and consequently oncogenic transformation [284,290].

To date, ten HBV genotypes (A–J) have been described, and increasing evidence suggest that genotypes can influence mutational patterns, development of vaccine escape mutants, and response to antiviral therapy [290]. Molecular epidemiological studies of HBV genotypes among different populations demonstrate that genotypes B and C predominate in East Asian countries, genotypes A and D in Europe and India, genotypes A, D, and E in Africa, and genotypes A, G, and H in the U.S. [290,291].

### 6.2. Direct Mechanism of HBV Oncogenesis

A direct correlation between HBV viral factors and development of HCC, even in the absence of cirrhosis, has been demonstrated [292]. These viral factors include high levels of viral replication [293], viral genotype [294,295], and specific mutations [295,296]. HBV encodes several proteins that exhibit direct oncogenic activities like growth factor-independent proliferation, resistance to growth inhibition, cellular migration and metastasis, angiogenesis, resistance to apoptosis, and the induction of oxidative stress [284]. Evidence suggests that surface and HBV x proteins play crucial roles in the pathogenesis of HBV-induced HCC via modulation of numerous cellular processes [287,290]. 

The oncogenic nature of HBV is primarily attributed to its ability to insert viral DNA into host cellular genome at fragile sites prone to development of mutations, causing chromosomal instability and the alteration of host gene expression [284]. Integrated viral DNA has been detected in 80–90% of HBV-related HCC [281,297]. The most common integrated viral genes are *HBx* and *preS/S* genes [286]. Frequently altered human genes include *TERT*, *MLL4*, *CCNE1*, *NTRK2*, *IRAK2*, and *MAPK1*, which are important for various cellular processes, such as telomerase activity, cell cycle progression, cell proliferation, apoptosis, and stress response [284,298].

Viral genome integration induces mutations in the *HBx* gene, which is strongly correlated with oncogenic transformation. HBx-mediated transcriptional regulation is a key mechanism in HBV-induced hepatocarcincogenesis. HBx functions as an oncogenic protein directly by binding to the proteins of transcription complex or indirectly by enhancing the expression of transcription factors [299] Consequently, HBx is involved in regulating DNA repair [287], expression of miRNAs [300], autophagy [281], cell proliferation and invasion [301], cell cycle progression [301], and angiogenesis [284]. Several targets of HBx include, tumor suppressor gene p53, DNMT, Wnt/β-catenin, and multiple transcription factors, such as E2F1, NF-κB, and AP-1 [299,302].

Accumulation of DNA damage is one of the driving mechanisms of mutation development and tumor formation. In vitro studies demonstrated higher levels of 8-hydroxydeoxyguanosine (8-OHdG) and lower levels of human DNA glycosylase alpha in HBx-expressing cells [303]. 8-OHdG is a molecular indicator of oxidative stress, which is known to induce DNA damage, while DNA glycosylase alpha is involved in base excision repair [287]. These results indicate that HBx promotes genetic mutations and interferes with DNA repair mechanisms, both of which are hallmarks of carcinogenesis.

Mutations in the tumor suppressor protein p53 have been implicated in nearly all types of cancer. The function of p53 is critical to the preservation of genomic integrity. The expression of p53 is upregulated in response to cellular stress, leading to induction of cell cycle arrest to allow for DNA repair and p53-dependent apoptosis [304]. HBx inactivates p53 directly by binding the protein and indirectly by inducing an inactivating mutation in the *p53* gene [290]. The loss of p53 creates a tolerant environment for the proliferation of mutant cells and malignant transformation.

Increasing evidence suggests that autophagy is involved in HBV-related HCC [281]. Proper autophagic response is important in preserving genome integrity by degrading damaged cells. *Atg5*, *Atg7* and *Beclin 1* are genes involved in the regulation of autophagy, and suppression of these genes inhibits the autophagic response [305]. Using models of knockout autophagy-related genes, several studies have demonstrated that autophagy plays a role in promoting HBV DNA replication [306,307]. Sir et al., further showed that HBV core protein and autophagosome colocalize in HBV-positive cells, indicating that the autophagosome may act as a docking site for viral replication [306]. HBV mediates autophagic activities primarily through HBx [281,308,309]. Beclin 1 complexes with phosphatidylinositol 3-kinase catalytic subunit type 3 (PI3KC3) to mediate the formation of phosphatidylinositol 3-phosphate (PI3P), which is a crucial signaling molecule in the initiation of autophagy [309]. HBx induces autophagy by upregulating mRNA expression of Beclin-1 and activating PI3KC3 enzymatic activity [281]. Death-associated protein kinase (DAPK) is another protein that can induce autophagy through Beclin-1-dependent activation of PI3KC3 [310], and DAPK is activated by HBx [308].

Although the relationship between autophagy and HBV viral replication has been elucidated, the role of autophagy in HBV-related HCC remains unclear. Autophagy has been shown to promote oxidative stress and DNA damage in mice, resulting in the initiation of HCC [307]. Furthermore, mice with the deletion of *Atg5* and *Atg7^−/−^* genes develop multiple liver tumors [311]. However, *Beclin-1* gene expression is enhanced in early and intermediate stages of viral hepatitis, but is downregulated in HCC, suggesting that autophagy suppresses tumorigenesis [312]. These results suggest that high autophagy occurs in early liver disease and shifts to low autophagy after initiation of HCC. The mechanism by which the transition from high to low autophagy takes place remains unknown [281].

HBV surface proteins (preS and S) also exhibit oncogenic potential. A meta-analysis by Liu et al., demonstrates that infection with preS/S mutants increases the risk of HCC by 3.77-fold [313]. Mutated preS/S are produced by integration of defective viral sequences [314]. PreS/S mutants play a function in the induction of endoplasmic reticulum stress, an event that promotes oxidative DNA damage, and activation of signal transduction pathway that is responsible for cell cycle progression, cell proliferation, and anchorage-independent growth [315].

### 6.3. Indirect Mechanism of HBV Oncogenesis

Chronic inflammation is linked to several critical events that are involved in tumor initiation and promotion. Tumor-associated macrophages (TAMs) and T cells are the most common cell types found in tumor tissues [316]. Pro-tumorigenic roles have been demonstrated for all T cell subsets, except NK T cells [317,318,319]. In addition, mast cells, B cells, and neutrophils also contribute to tumorigenesis [316]. Research has suggested that the inflammatory microenvironment enhances mutation development. Inflammatory cells are sources of reactive oxygen species (ROS) and reactive nitrogen intermediate (RNI), which have been linked to the promotion of genomic instability [316]. Furthermore, inflammatory cells induce epithelial cells to produce ROS via the action of cytokines and chemokines [316].

**Figure 5:**
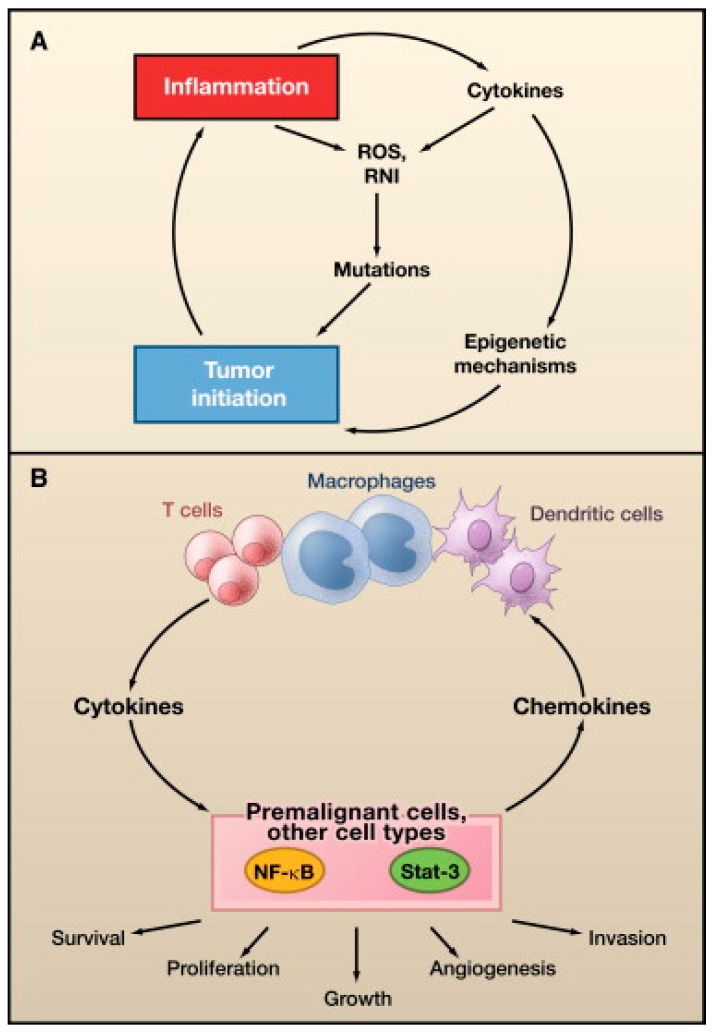
Role of Inflammation in Tumor Initiation and Promotion. (**A**) Tumor initiation. Reactive oxygen species (ROS) and reactive nitrogen intermediates (RNI) produced by inflammatory cells may cause mutations in neighboring epithelial cells. Also, cytokines produced by inflammatory cells can elevate intracellular ROS and RNI in premalignant cells. In addition, inflammation can result in epigenetic changes that favor tumor initiation. Tumor associated inflammation contributes to further ROS, RNI, and cytokine production; (**B**) Tumor promotion. Cytokines produced by tumor-infiltrating immune cells activate key transcription factors, such as NF-κB or STAT3, in premalignant cells to control numerous protumorigenic processes, including survival, proliferation, growth, angiogenesis, and invasion. As parts of positive feed-forward loops, NF-κB and STAT3 induce production of chemokines that attract additional immune/inflammatory cells to sustain tumor-associated inflammation. Reproduced with permission from Grivennikov, S.I., Immunity, Inflammation, and Cancer; published by *Cell*, 2010.

NF-κB and STAT3 are major pro-inflammatory and pro-tumorigenic signaling pathways that are involved in the development of many cancers (Figure 5) [284,316]. It has been suggested that NF-κB and STAT3 signaling pathways are activated in HBV-related HCC by inflammatory cytokines and HBx [316,320]. Some of the most potent activators of NF-κB and STAT3 include viral DNA and RNA that activate Toll-like receptors (TLRs), tumor necrosis factor (TNF), and interleukin (IL)-1 [320]. One of the most crucial NF-κB-dependent cytokine is IL-6 [321]. IL-6, in turn, is a key STAT3 activator, and the activation of STAT3 has been shown to play a prominent role in cell survival and proliferation, tissue invasion, angiogenesis, and promotion of HCC development [316,321,322].

### 6.4. Therapeutic Options

One of the challenges in treating HCC is that the majority of HCC cases develop in the background of a cirrhotic liver, and therapies such as radiation and chemotherapy damage surrounding normal hepatocytes, which can exacerbate liver failure [323]. Surgical resection offers the best results, but this is possible in less than 5–10% of all cases [323,324]. Resection is a potential option in patients whose tumor is confined to a portion of the liver, whose liver function tests are mostly normal, and advanced cirrhosis or active hepatitis are not present since the risk of hepatic decompensation is much higher in patients with cirrhosis [323,325]. The five-year survival rate after surgery as high as 70%, but recurrence develops in more than 30% of patients [325].

For patients with decompensated cirrhosis whose tumor meets the Milan criteria for HCC (single lesion <5 cm or up to 3 lesions with each <3 cm), liver transplantation is the preferred treatment modality, with a five-year survival of up to 75% [323,325]. Other available therapies for small, localized tumors that are not resectable include local ablative therapies, such as transarterial chemoembolization (TACE), percutaneous ethanol injection (PEI), microwave coagulation, radiofrequency ablation, and photodynamic therapy [323,324]. These options can also be used for patients with advanced HCC with involvement of both lobes of the liver or as palliative therapy for those with distant metastasis [323].

Sorafenib is one of the two approved systemic chemotherapies for the treatment of advanced HCC. Sorafenib is a multikinase inhibitor that targets VEGF receptor, platelet-derived growth factor receptor, RET, c-KIT, and Ras/MAPK pathway, among others [325]. These kinases play important roles in tumor proliferation and angiogenesis [325]. Two phase III randomized placebo-controlled trials, the SHARP trial [326] and a similar trial in Asia [327], reported median overall survival of 10.7 and 6.5 months with sorafenib, respectively.

The FDA recently approved a second systemic chemotherapy for the treatment of patients with HCC. A phase III study of regorafenib for the treatment of HCC in patients whose disease progressed on sorafenib demonstrated significant improvement in overall survival of 10.6 months compared to 7.8 months for patients in the placebo arm and significant improvement in progression-free survival of 3.1 and 1.5 months for the regorafenib and placebo arm, respectively [328]. In April 2017, regorafenib was approved as a second-line treatment for advanced HCC.

Lifelong antiviral therapy has been shown to improve the overall survival and reduce HCC recurrence after curative HCC treatment [329]. The goals of treatment in chronic HBV infection are to suppress viral replication, to prevent disease progression, and to reduce the risk of HCC recurrence after curative HCC treatment. The indications for treatment of chronic HBV are based on HBV DNA > 2000 IU/mL, ALT > upper limit of normal (ULN) and/or at least moderate liver fibrosis [330,331]. Patients with cirrhosis, with any detectable HBV DNA level and regardless of ALT levels, should be treated. Patients with HBV DNA > 20,000 IU/mL and ALT level greater than 2 times the upper limit of normal should be treated regardless of the degree of fibrosis, which can be determined by liver biopsy or non-invasive testing [330,331]. Additional patient groups in whom treatment should be considered are pregnant women with high viral loads to prevent vertical transmission and immunocompromised patients, to prevent HBV reactivation [331].

Current approved drugs for chronic HBV infection include pegylated interferon (IFN) α and nucleos(t)ide analogues (NAs). Pegylated IFN-α works by inducing long-term immunological response. The disadvantages of pegylated interferon are a highly variable response rate and its potential side effects, limiting its uses among many patient populations [331,332]. NAs work by inhibiting viral replication and can be divided into those with low barrier against HBV resistance (lamivudine, adefovir, telbivudine) and those with high barrier to HBV resistance (entecavir, tenofovir disoproxil fumarate, tenofovir alafenamide) [331,332,333]. Lamivudine, adefovir, and telbivudine are not recommended in the treatment of chronic hepatitis B since they can potentially increase the risk of HCC in cases with drug-resistant strains [314]. NAs with a high barrier to resistance have the advantages of predictable high long-term efficacy and a favorable safety profile [331,332,333]. In addition, NAs are the only treatment option for those with decompensated liver disease, liver transplants, extrahepatic manifestations, acute hepatitis B, or severe chronic HBV exacerbation, as well as for the prevention of HBV reactivation in immunocompromised patients [333,334,335]. Combination therapies are not recommended due to the lack of evidence for superiority [331].

## 7. Hepatitis C Virus

Hepatitis C virus belongs to the *Hepacivirus* genus and *Flaviviridae* family. Like HBV, HCV affects the liver and can cause acute and chronic infection. Approximately 75–85% of people will progress to chronic HCV infection, and the rate of progression depends on several factors [336]. Risk factors for chronic HCV infection include, an age greater than 25 years at the time of infection, male gender, African American, asymptomatic acute infection, HIV coinfection, and immunocompromised status [336].

Various reports have estimated that up to 170 million adults worldwide are chronically infected with HCV [337,338,339], and 4 million new cases are diagnosed annually [340]. HCV is transmitted through the same routes as HBV, with parenteral contact being the most common route. Populations at high risk of HCV infection are those who received blood transfusions prior to 1990, intravenous drug users, intranasal cocaine users, people with tattoos and body piercings, healthcare workers, dialysis patients, and those participating in high risk sexual activities [340].

In contrast to developing countries where HBV is the leading cause of HCC, chronic HCV infection is the major risk factor for HCC in developed countries [339]. HCV accounts for up to 25% of cases of HCC in Asia and Africa, and 60% in the U.S. [284,285]. Whereas, the carcinogenic effect of HBV is primarily attributed to the direct effects of viral proteins, inflammation appears to be the major mechanism of HCV-induced hepatocarcinogenesis. The annual incidence of HCC in patients with advanced liver cirrhosis is very high (1–7% per year), and a sustained virological response does not eliminate the risk of HCC development in those who have already developed cirrhosis [339,341].

HCV has been linked to several other malignancies, such as carcinomas of the head and neck, biliary duct, bladder, renal, pancreas, thyroid, breast, and prostate, as well as B-NHL, but the role of HCV in these malignancies is still under investigation [342]. Among B-NHL, HCV infection is particularly linked to marginal zone lymphoma (MZL) and diffuse large B-cell lymphoma (DLBCL) [343]. MZL accounts for 12% of all B-NHL, and is most commonly seen in patients 65 years or older [343]. DLBCL is responsible for 30–58% of all B-NHL, and has a predilection for elderly males [344]. The oncogenic mechanism in HCV-associated B-NHL remains unclear. More recent studies have implicated Th2 cytokines, characterized by IL-4, IL-6, IL-10, and TNF-α, in the pathogenesis of B-cell lymphomas [345,346].

### 7.1. HCV Genome

HCV is an enveloped, single-stranded, positive-sense RNA virus of 9.6 kb genome size. The viral genome encodes for structural (Core, E1, E2) and non-structural (NS2, NS3, NS4A, NS4B, NS5A, and NS5B) proteins [284]. Unlike HBV, the integration of viral genome into host cell genome is very limited in HCV infection. The majority of HCV life cycle is spent in the cytoplasm, where viral replication machinery interacts closely with the ER membrane, mitochondrial outer membrane, and lipid droplets [347]. Seven genotypes of HCV (genotype 1–7) have been recognized, and are clinically important due to their different susceptibility to interferon therapy [347].

### 7.2. Direct Mechanism of HCV Oncogenesis

**Figure 6:**
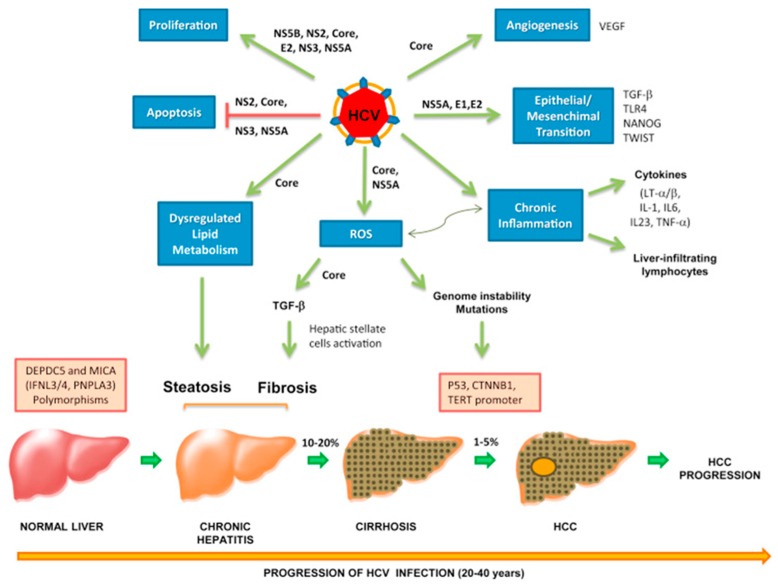
Hepatitis C virus (HCV)-related mechanisms of carcinogenesis: from HCV infection to hepatocellular carcinoma (HCC). Chronic HCV and associated liver cirrhosis represent major risk factors for HCC development. Hepatocarcinogenesis is a multistep process that may last for years; it involves progressive accumulation of different genetic alterations which lead to malignant transformation. Malignant transformation of hepatocytes occurs through increased liver cell turnover, induced by chronic liver injury and regeneration, in the context of inflammation and oxidative stress. HCV proteins may directly upregulate mitogenic pathways, block cell death and induce reactive oxygen species (ROS) production. Moreover, HCV triggers persistent inflammation with accumulation of liver-infiltrating lymphocytes and production of several cytokines, such as LTα and LTβ, which are tightly linked to HCC development. Chronic inflammation exacerbates ROS production, which is considered a main source of genetic mutations. ROS are also associated with TGF-β pathway induction, leading to hepatic stellate cell activation and fibrogenesis. Transforming growth factor-β (TGF-β), together with TLR4, plays an important role in the epithelial–mesenchymal transition. HCV dysregulates host lipid metabolism, causing liver fat accumulation which in many patients is associated with HCC. HCV is also able to induce angiogenic and metastatic pathways. Polymorphisms, mainly in DEPDC5 and MICA genes, have been recently shown to increase the risk of developing HCC. HCC, hepatocellular carcinoma; HCV, hepatitis C virus; ROS, reactive oxygen species; TGF, transforming growth factor. Reproduced with permission from Vescovo, T., Molecular mechanisms of hepatitis C virus–induced hepatocellular carcinoma; published by *Clin. Microbiol. Infect.*, 2016.

Evidence suggests that core NS3, NS4B, and NS5A proteins promote tumorigenesis in vivo through the direct interaction with host cell factors that are involved in apoptosis, DNA replication, DNA repair, cell cycle progression, and angiogenesis [338,348] (Figure 6). HCV core protein is essential for the assembly of infectious virions, transcriptional regulation, apoptosis, lipid metabolism, and cell transformation [349]. The NS3 protein, together with its cofactor NS4A, functions as a helicase and protease to cleave polypeptides into viral proteins [350]. NS4B is a key scaffold protein in the formation of viral replication complex [351]. The NS5A protein is composed of three domains. NS5A domains I and II participate in RNA replication while domain III assists in virus assembly [352]. Although not directly oncogenic, NS5B, an RNA-dependent RNA polymerase, is a crucial component of the HCV replicase complex and plays an important role in the tumorigenic pathway [352].

Over-expression of HCV viral proteins has been demonstrated to promote cellular proliferation, transformation, and tumor initiation, suggesting the direct role of HCV proteins in activating tumorigenic pathways [349,353]. HCV core protein, NS4B, and NS5A have been shown to activate Wnt/β-catenin signaling pathway in Huh7 cells, which is a well-differentiated hepatocyte derived carcinoma cell line [349,354]. Aberrant activation of the Wnt/β-catenin signaling pathway is closely associated with tumor cell growth, metastasis, and recurrence of HCC [355].

Tumor suppressor proteins, such p53, p21, p73, Rb, ATM, and nibrin (NBS1), are inhibited by HCV viral proteins [302,339,347]. Rb protein regulates cell cycle progression by repressing E2F, a transcription factor that is essential for entry into S phase of the cell cycle. HCV NS5B binds to and inhibits Rb, and thus allows for transformed cells to enter the cell cycle unchecked [356]. HCV NS3/4A protein interacts with ATM, a protein that detects double-strand DNA breaks, and impairs DNA repair processes, which render the cell more sensitive to DNA damage [357].

The impact of HCV viral proteins on the activity of p53 is a topic of debate. Some have reported that low levels of HCV core protein stimulate expression of p53, while others have reported that high levels of core protein suppress transcriptional activity of p53 [356]. These discrepancies suggest that core protein and p53 have a viral concentration-dependent relationship, but whether it is a direct or inverse relationship is unclear. The role of HCV non-structural proteins in the regulation of p53 appears to be primarily inhibitory. NS3/4A and NS5A induce the relocalization of p53 from nucleus to cytoplasm, thereby interfering with p53-induced apoptosis [356]. Another mechanism by which HCV mediates aberrant cellular survival is through the inhibition of TNF-α-mediated apoptosis signaling. NS5A prevents TNF-α-mediated apoptosis by blocking the activation of caspase-3, which is highly expressed in apoptotic cells [358].

Oxidative stress is known to induce DNA damage and activate oncogenic signaling pathways [359]. In the liver, ROS is mainly generated by the mitochondria [347]. Given that HCV replication is closely associated with the mitochondrial membrane, a strong correlation exists between oxidative stress, mitochondrial injury, and DNA damage. HCV core protein and NS5A are key players in the induction of oxidative stress. Enhanced expression of HCV core protein is associated with mitochondrial injury, increased ROS levels, and increased lipid peroxidation products [347]. NS5A induces oxidative stress by disrupting intracellular calcium homeostasis, leading to cell death via mitochondria-mediated and NF-κB and STAT-3-mediated apoptotic pathways [347]. Increased oxidative stress further activates transforming growth factor-β (TGF-β), a major profibrogenic cytokine [360]. Moreover, the expression of TGF-β is upregulated by HCV core protein, providing a more direct role of HCV protein in fibrogenesis [347].

Viral proteins also play a direct role in the induction of angiogenesis. For example, HCV core protein upregulates hypoxia inducible factor 1α, which in turn stimulates the expression of VEGF, cyclooxygenase 2, and MMPs [361,362]. These are important mediators of endothelial cell growth and vascular formation, and are potential therapeutic targets.

### 7.3. Indirect Mechanism of HCV Oncogenesis

As opposed to HBV infection, in which viral proteins are the primary players in oncogenesis, the oncogenic properties of HCV have been attributed primarily to indirect pathways via inflammation and oxidative stress [284] (Figure 6). This notion is supported by the direct correlation between the degree of fibrosis and the increasing risk of HCC in chronic HCV infection [339]. HCV viral proteins promote the release of profibrogenic cytokines and chemokines, such as TGF-β [363]. The induction of profibrogenic mediators is regulated by pathways such as ROS production, MAPK, NF-κB, and PDGF pathways [339]. It has been suggested that TGF-β plays dual functions in cancer. TGF-β displays tumor suppressor activity in normal livers and shifts to fibrogenic activity in chronic inflammation [356]. Furthermore, TGF-β can mediate immunosuppression, which allows for tumor cells to escape detection by the immune system and may be an early step in the hepatocarcinogenesis pathway [284].

T-helper (Th) 1 inflammatory cells, characterized by IFN-γ and IL-2, predominate in the liver during chronic HCV infection and contribute to low-grade liver inflammation [364]. Other inflammatory cytokines, such as TNF-α, IL-1, IL-23, IL-6, and lymphotoxin, have been implicated in chronic liver inflammation and HCC development [356]. Lymphotoxin, in particular, plays a critical role in the progression of HCC. Research has demonstrated that HCV viral proteins, especially NS5B, and host immune cells can directly stimulate lymphotoxin release in a human hepatocyte cell line [284]. Hepatic lymphotoxin overexpression in mice leads to chronic progressive hepatitis, hepatotoxicity, and spontaneous HCC formation [365]. Experiments further suggest that the activation of NF-κB by lymphotoxins may be the triggering event in HCV-related hepatocarcinogenesis by causing a vicious cycle of inflammatory cytokine production, cell death, and malignant transformation [365].

Host factors, such as metabolic alterations, are closely associated with increased risk of HCC development [284]. Diabetes mellitus and obesity have been shown to synergistically promote liver inflammation and carcinogenesis in association with HCV infection [366]. In a mouse model of obesity, CD8^+^ T cells and NK cells were found to modulate lipid metabolism in hepatocytes, which was directly correlated with steatosis, inflammation, and carcinogenesis [367]. Recent reports have demonstrated the role of HCV in the reprogramming of lipid metabolism by upregulating IL-1, IL-6, and TNF-α expression [284]. The accumulation of free fatty acids triggers the production of ROS as a consequence of mitochondrial injury and endoplasmic reticulum (ER) stress [356]. Oxidative stress can stimulate the inflammatory cascade leading to release of pro-inflammatory cytokines, promotion of collagen expression, which culminates in liver fibrosis, and the induction of gene mutations and chromosomal instability [356].

### 7.4. Therapeutic Options

The goals of treatment in HCV infection are to achieve a sustained virological response (SVR) and to reduce complications from chronic hepatitis, including HCC. For HCV-related HCC, treatment options are the same as those from HBV infection. SVR is defined as undetectable HCV RNA for at least 12 weeks following the completion of therapy [344].

Treatment is recommended for all patients, except for those with life expectancy of less than 12 months [368]. Highest priority for treatment should be given to those with advanced fibrosis, compensated cirrhosis, organ transplant recipients, type 2 or 3 cryoglobulinemia with end-organ manifestations, proteinuria, nephrotic syndrome, or membranoproliferative glomerulonephritis given significant risk of severe complications in these patient populations [368]. Additional groups of patients that should receive high priority for treatment, include those with fibrosis, HIV-1 coinfection, HBV coinfection, other coexistent liver disease, debilitating fatigue, type 2 diabetes mellitus, and porphyria cutanea tarda [368].

Direct acting antiviral (DAA) agents and IFN-based therapies are available treatments in chronic HCV infection. Pegylated IFN-α plus ribavirin is an older regimen that is less commonly used today due to its toxicities. Antineoplastic properties of IFN are mediated through their inhibitory effects on hepatic stellate cell-mediated fibrogenesis and on inhibitors of MMP, resulting in decreased fibrogenesis and increased fibrinolysis, respectively [369,370]. In addition, IFN works by inhibiting angiogenesis, promoting tumor cell apoptosis, and stimulating hepatic stellate cell death [370]. Response to IFN-based therapy is partly dependent on genotype. Approximately 80% of patients with genotypes 2 and 3 achieved SVR, while only 50% of patients that were infected with genotypes 1 and 4 achieved SVR [371]. Genotype 5 and 6-infected patients have intermediate response rates [371]. While limited by its toxicities, IFN remains an important adjunct to DAAs in difficult-to-cure patients, such as patients with genotype 3 infection [372].

Currently, DAA agents are first-line therapies due to higher efficacy in achieving virologic cure and fewer serious adverse events. Cure rates have risen from near 50 to over 90% in most groups of patients with the use of DAAs [370]. Moreover, liver related complications, such as hepatic encephalopathy and variceal hemorrhage, are reduced and hepatic function, as indicated by the Model for End-Stage Liver Disease (MELD) score is improved [370]. However, the limited availability and high costs of DAAs restrict their use to mainly high-income countries [372]. Treatment options are based on HCV genotype, history of prior therapy, and presence of cirrhosis (Table 1) [368,373]. In general, patients with cirrhosis require longer duration of therapy and addition of ribavirin. For patients who have failed prior therapies, treatment regimens are more limited but dosage and duration of therapy are similar to treatment-naïve regimens.

## 8. Human T-Cell Lymphotropic Virus-1

Human T-cell lymphotropic virus 1 (HTLV-1) is a delta RNA retrovirus that was first isolated in 1979 [374]. HTLV-1 most notably causes the peripheral T cell neoplasm adult T-cell lymphoma (ATLL) [42,375], and is also associated with polymyositis, HTLV-1-associated myelopathy/tropical spastic paraparesis (HAM/TSP) [376], infective dermatitis associated with HTLV-1 (IDH) [377], arthropathy, Sjögren’s syndrome, and facial nerve paralysis [378]. There are an estimated 5–20 million HTLV-1 carriers worldwide [379], and approximately 3–5% of carriers develop ATLL [380,381]. HTLV-1 is endemic to Japan, Iran, Caribbean Islands, Honduras, Brazil, Peru, Ecuador, Papua New Guinea, and West Africa [382,383]. Prevalence is 1–5% in these regions, but this is likely an underestimation [379,382,383]. Estimates are based primarily on seroprevalence of blood donors, hospitalized, and pregnant patients, so the results may not accurately represent the entire population of these regions [379]. Seroprevalence in the United States is only 0.018% [384].

HTLV-1 can be transmitted through breast milk, sexual intercourse, and infected blood products [385]. Transmission via breastmilk occurs 20.3% of the time that an infected mother nurses the child for at least six months and 7.4% of the time when the mother nurses the child less than six months [386]. Male to female is the most common form of sexual transmission, with a much lower rate for female to male [387,388]. Donor blood transmission is highly uncommon due to the screening of donor blood in many countries, including the United States, Japan, Brazil, France, and the United Kingdom (UK) [389]. HTLV-1 has a long latency period, and ATLL typically occurs 40–60 years after initial infection [390]. In 2008, there were 2100 worldwide cases of ATLL, with most occurring in endemic regions [7]. ATLL occurs between age 20 and 80 years, with an average onset age of 58 [391]. ATLL has infrequently been observed in children and adolescents. Due to the long latency period of HTLV-1, ATLL generally results from infection that is acquired during breastfeeding early in life [392,393].

**Figure 7:**
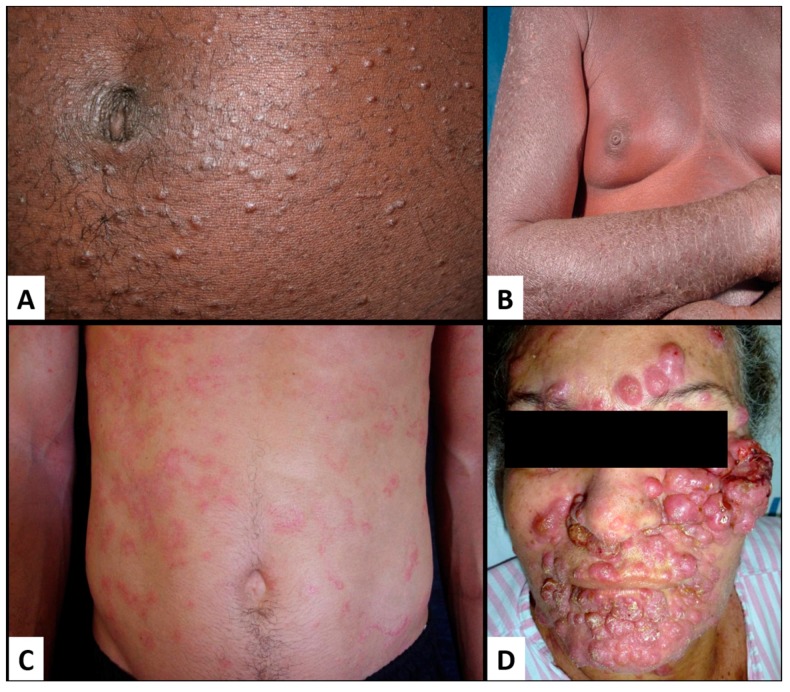
Examples of cutaneous lesions observed in ATL. (**A**) Chronic form with papular pattern; (**B**) Acute form showing exfoliative erythroderma; (**C**) Smoldering form with a pattern of papules and erythematous scaly plaques; (**D**) Primary cutaneous tumoral form. Reproduced with permission from Oliveira P.D., Adult T-cell leukemia/lymphoma; published by *Rev. Assoc. Med. Bras.*, 2016.

ATLL is a clonal proliferation of CD4 T regulatory cells and exists as four different subtypes in the Shimoyama classification: acute, lymphoma, chronic, and smoldering [394] (Table 2). Classification is based upon organ involvement, leukemic manifestation, lactate dehydrogenase (LDH) level, and calcium level [394,395]. The acute type is the most common, and comprises of approximately 60% of cases, with lymphoma, chronic, and smoldering subtypes comprising 20%, 10%, and 10% of cases, respectively [396]. The acute and lymphoma subtypes are considered to be aggressive and have a large tumor burden, lymph node, and blood involvement, and hypercalcemia [394,397]. Overall survival (OS) rates are 8.3 months for the acute subtype and 10.6 months for the lymphoma subtype [396]. The chronic and smoldering subtypes are considered indolent, with OS rates of 31.5 months and 55 months, respectively [396]. Clinical features of ATLL include generalized lymphadenopathy, splenomegaly, hepatomegaly, skin lesions, hypercalcemia, lytic bone lesions, involvement of central nervous system and gastrointestinal tract [398], and opportunistic infections that are caused by Pneumocystis jirovecci, candida, cytomegalovirus, and *Strongyloides stercoralis* [399]. Skin lesions are of a wide variety, including rashes, plaques, papules, and erythrodermic and purpuric lesions [400] (Figure 7).

### 8.1. HTLV-1 Molecular Biology

HTLV-1 has a single-stranded RNA genome of about 9 kb [401]. The positive-sense strand encodes the usual retroviral structural gene products *Gag* (capsid, nucleocapsid, matrix), Pro, *polymerase* (Pol), and *Env* from unspliced or singly spliced transcripts [402,403,404]. Two long terminal repeats (LTRs) flank the structural genes [401]. The pX region at the 3′ end of the genome encodes several accessory genes, including *Tax*, *Rex*, *p12*, *p30*, *p13*, and *HTLV-1 bZIP factor* (*HBZ*) [405,406,407,408]. Upon HTLV-1 cell entry and uncoating, the viral genome undergoes reverse transcription into DNA before integrating into the host genome as a provirus [409]. Integration often occurs near transcription factor binding sites, including those for STAT1, TP53, and HDAC6 [410,411]; this is 15 kb upstream of host genes that are commonly dysregulated in leukemias [412].

HTLV-1 has the ability to infect CD4 cells, CD8 cells, and dendritic cells [401]. Viral cell-attachment and spread within the host require interaction of multiple proteins and processes. *Env* encodes a precursor protein, which is cleaved into two proteins that are essential for viral infectivity: transmembrane (TM) and surface (SU) protein. TM and SU are acquired by the virions as they are released from the infected cell [413,414]. SU binds to the host cell surface receptors glucose transporter 1, neuropilin-1, and heparan sulfate proteoglycans, causing viral-cell membrane fusion and subsequent infection [415,416,417,418,419,420].

HTLV-1 infection primarily spreads cell-to-cell within the host via two mechanisms: mitosis and viral synapse transfer [421,422]. In mitosis, daughter cells contain proviral DNA that is inserted into the host genome at the same site as in the parent cell [421]. During non-mitotic cellular transfer, cell-to-cell contact is required because HTLV-1-positive cells rarely release free virions, and only 1 of 10^5^ to 1,000,000 of released virions are infectious [423,424]. Cell-to-cell transmission is 10,000 times more efficient than virion infection of a non-neighboring cell [425] and explains why cell-containing fluids, such as blood, semen, and breast milk are successful in transmitting infection [415].

When an infected cell contacts an uninfected cell, Gag and Env proteins polarize to the area of contact to facilitate fusion [422]. The cell transiently expresses high levels of Tax protein and intercellular adhesion molecule-1 (ICAM-1), forming a virological synapse (VS) [426] or a viral biofilm [410]. Virions released from the infected cell enter the VS and bind HTLV-1 receptors before entering the target cell [413,414]. Once inside of the target cell, HTLV-1 will form a provirus and integrate into the host genome at a site unique from that of the source cell, creating a new clone [421].

### 8.2. HTLV-1 Oncogenic Proteins 

Tax is an HTLV-1 transactivator protein that is coded by the sense strand of the pX provirus region [427,428]. Tax plays an important yet complicated role in ATLL development, as it interacts with more than a hundred cellular proteins to prevent apoptosis, enhance cell signaling, induce cell cycle dysregulation, activate proto-oncogenes, and interfere with DNA repair [429,430,431]. Tax interacts with factors such as AP-1, NF-κB, CREB/ATF, CBP/p300, and p300/CBP-associated factor (P-CAF) serum responsive factor (SRF) to induce and repress transcription of certain genes [431]. Cytokines and receptors that are increased include IL-2/IL-2 receptor (IL-2R), IL-9, IL-13, and IL-15/IL-15R [401,432,433]. *p53*, *cyclin A*, and *c-myb* genes are repressed via Tax interaction with CREB/ATF [434,435,436]. Tax continually activates the NF-κB pathway, which can lead to unregulated lymphocyte development. Tax also enhances the G1/S transition via activation of cyclin D/CDK [401] and the G0/G1 transition via hyperphosphorylation of hDLG [429]. Not only does Tax affect transcription and cell-cycle regulation, it also contributes to genetic damage. This damage occurs directly through clastogenic DNA damage and aneuploidic effect, and indirectly through the over-activation of the DDR pathway [431]. Although Tax appears as essential for initiating ATLL transformation, it is repressed after tumor initiation and is only detected in approximately 60% of ATLL circulating cells [432,437]. The effects of Tax, however, are seen even after its repression [438].

HBZ is encoded by the antisense strand of the HTLV-1 provirus pX region [405,406,407,408], and unlike Tax, is present in 100% of ATLL cells [439]. Survival of HBZ in ATLL is likely due to its low immunogenicity, whereas Tax is highly immunogenic and the target of a cytotoxic T lymphocyte (CTL) response [408,427]. Although HBZ enhances proliferation of T cells, it is unexpectedly antagonistic to many oncogenic functions of Tax, including activation of classical NF-κB pathway, AP-1, NFAT, and CREB [440,441]. Repression of Tax function by HBZ may allow for HTLV-1 to evade the immune system and develop ATLL [442].

HBZ stimulates lymphocyte proliferation through upregulation of *E2F1* gene [440] and prevents apoptosis through the inhibition of the pro-apoptotic *Bim* gene [408]. HBZ also counteracts senescence through the inhibition of the classical NF-κB pathway [408,443]. MicroRNAs induced by HBZ compromise host genomic integrity, thus contributing to ATLL transformation [398]. These various functions demonstrate the oncogenic importance of HBZ. Furthermore, HBZ levels correlate with PVL, which is the greatest risk factor for ATLL [444,445].

### 8.3. ATLL Transformation

ATLL cells are named flower cells due to their multilobulated nuclei and are usually CD4^+^CD8^−^ but can occasionally be CD4^+^CD8^+^ or CD4^−^CD8^+^. The tumor cells also frequently express CD25, CD2, CD3, CD5, CCR4, and FoxP3, but usually lack CD7 [446,447]. ATLL is diagnosed when there is histological evidence of T lymphoid malignancy and detectable HTLV-1 antibodies [447]. The greatest risk factor for developing ATLL is a high HTLV-1 proviral load (PVL) (>4 copies/100 peripheral blood mononuclear cells) [448]. PVL is primarily determined by HLA subtype [449], and is established early after infection. PVL remains stable over many years in most HTLV-1 carriers [450]. Oligoclonal proliferation was previously thought to increase the risk of ATLL development, but it has been shown that the number of clones, not the degree of oligoclonality, increases the risk of ATLL development [390].

Multiple events must occur for HTLV-1 infected cells to develop into ATLL: HTLV-1-mediated proliferation, immune system evasion, and genetic alteration of growth potential [446]. This process is not yet completely understood. Two proteins appear as essential for HTLV-1 infection transformation into ATLL: Tax and HBZ. Tax is fundamental for initiating ATLL transformation by promoting cellular proliferation, genetic instability, and cell cycle dysregulation [432], whereas HBZ appears as necessary for the propagation of the transformed cell line [439]. Although chromosomal abnormalities appear in virtually all ATLL, a specific combination or amplification/deletion events have not yet been identified. Known alterations at the genetic level tend to occur near genes that regulate the G1/S transition [451], including CKD inhibitors, such as p15 (INK4A), p16 (INK4B), p18 (INK4C), p19 (INK4D), p21 (WAF1), p27 (KIP1), and p57 (KIP2), as well as p53 and Rb [451]. p53 mutations occur in 10–50% of ATLL, which is higher than in indolent ATLL [452,453]. Rb tumor suppressor is absent in 50% of ATLL, but the Rb gene is uncommonly altered in structure [453,454,455].

Epigenetic abnormalities, including alteration in DNA methylation patterns and histone modification, also play a role in ATLL transformation [398]. Specifically, polycomb repressive complex 2 (PRC2) and EZH2 exhibit an increased activity in ATLL, which leads to the accumulation of H3K27 me3. H3K27 me3 suppresses miRNAs, tumor suppressors, epigenetic modifiers, and transcription factors [398,438].

### 8.4. Therapeutic Options

**Figure 8:**
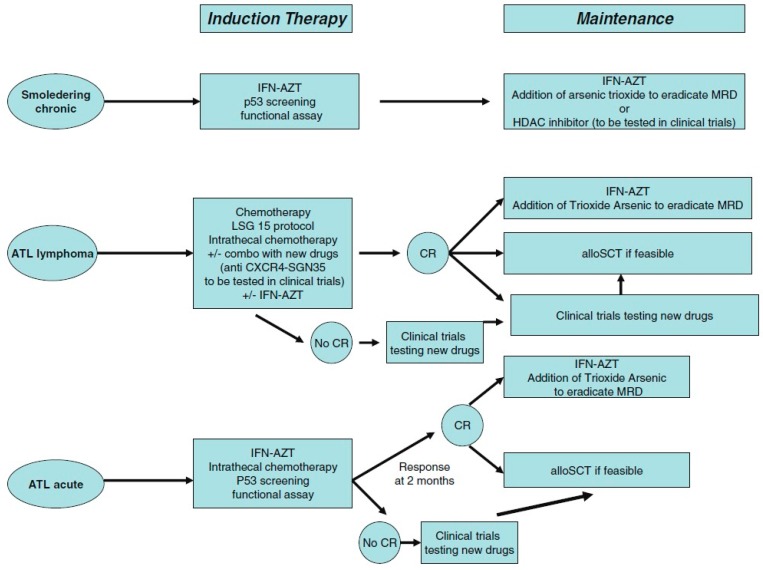
Recommended treatment strategy for patients with acute, lymphoma or chronic and smoldering subtypes of adult T-cell leukemia/lymphoma (ATL). CR: complete remission, MRD: minimal residual disease, AZT: zidovudine, IFN: interferon alpha, alloSCT: allogeneic stem cell transplantation, HDAC: histone deacetylase. Reproduced with permission from Marçais, A., Therapeutic options for adult T-Cell leukemia/lymphoma; published by *Curr. Oncol. Rep.*, 2013.

ATLL does not have a high response rate to treatment. ATLL treatment is guided by ATLL Shimoyama classification [447] (Figure 8). Clinical response criteria have been defined to assess therapies and guide treatment algorithms [456]. A complete response (CR) is defined as: all measurable tumor lesions, including lymphadenopathy, are undetectable and the absolute lymphocyte count has normalized to <4 × 10^9^ cells/L [456,457]. An unconfirmed CR is a 75% reduction of tumor size and an absolute lymphocyte count of less than 4 × 10^9^ cells/L, and a partial response is a 50% reduction in absolute lymphocyte count and tumor size [456,457].

The original mainstay treatment for ATLL was chemotherapy based upon Japan Clinical Oncology Group (JCOG) recommendations [447]. Common chemotherapy regimens include VCAP-AMP-VECP, which are comprised of vincristine, cyclophosphamide, doxorubicin, and prednisolone (VCAP); doxorubicin, ranimustine, and prednisolone (AMP); and, vindesine, etoposide, carboplatin, and prednisolone (VECP), or CHOP (doxorubicin, cyclophosphamide, vincristine, and prednisone) [395]. Antiviral therapy is also now being used. Meta-analysis has shown zidovudine (ZDV) and IFN-α to be effective treatments for chronic and smoldering subtypes, as well as a subset of acute subtypes [458]. Arsenic can act as an adjunct therapy to IFN to induce cell cycle arrest and apoptosis in HTLV-1 and ATLL cells [459,460,461]. Data are preliminary but suggest that arsenic could also be paired as part of a triple maintenance therapy with ZDV and IFN after successful induction therapy. This therapy is thought to help transform an immunodeficient T regulatory and Th2 phenotype to an immunocompetent Th1 phenotype [462]. Monoclonal antibodies, such as an anti-CD25 antibody [463,464] and mogamulizumab, an anti-CCR4 antibody [465,466], have proven successful as ATLL treatment in clinical trials, whereas, A24, an anti-transferrin receptor antibody, has shown promise in preclinical studies [467]. Watchful waiting is an option for chronic and smoldering subtypes [398]. For treatment-refractory disease, allogeneic stem cell transplantation (alloSCT) is a potentially curative option [447].

Promising new therapies include vorinostat and romidepsin (histone deacetylase inhibitors), alemtazumab (anti-CD52 antibody), and brentuximab vedotin (anti-CD30 antibody) [468]. A recent animal model indicated that pulsed dendritic cell therapy may decrease PVL by stimulating HTLV-1 responsive cytotoxic lymphocytes [469]. A pilot clinical trial using belinostat in combination with AZT-based maintenance therapy for aggressive leukemic ATL types is currently underway (NCT02737046).

Adjunct treatments are also available to address hypercalcemia and opportunistic infections that are associated with ATLL. Hypercalcemia can be managed with hydration and bisphosphonates [447,468]. Opportunistic infections should be promptly treated, and prophylaxis for *Pneumocystis jirovecci* pneumonia, viral infections, and fungal infections is recommended with trimethoprim-sulfamethoxazole, valacyclovir, and anti-fungal agents, respectively [447,468]. Additionally, patients with history of exposure to *Strongyloides stercoralis* can be given ivermectin and albendazole for prophylaxis against systemic strongyloidiasis [447,468].

## 9. Conclusions

Viral oncology is a field of growing interest that is continually under research. Promising new agents are emerging for the treatment of virus-related cancers. As our understanding of the molecular pathogenesis linking oncovirus and human cancer continues to improve, we are seeing a push for the development of a multitude of potential treatments that may enhance the killing of infected tumor cells, while sparing normal, healthy cells, and thereby maximizing the therapeutic index. The seven known human oncoviruses target many of the same host signaling pathways to induce oncogenesis. Novel therapies that have shown efficacy in one virus-related cancer may be expanded to all virus-related cancers sharing the same pathways. These innovative approaches may finally put a cure for cancer on the horizon.

## Figures and Tables

**Table 1:** Recommended treatment options for HCV infection in treatment-naïve patients based on EASL and AASLD/IDSA treatment guidelines.

Genotype	Treatment Options	Duration of Treatment	Strength of Evidence
Genotype 1a without/with compensated cirrhosis	Ledipasvir/sofosbuvir	12 weeks	I, A
	Velpatasvir/sofosbuvir	12 weeks	I, A
	Grazoprevir/elbasvir	12 weeks	I, A
	Paritaprevir/ritonavir/ombitasvir/dasabuvir ± ribavirin	12 weeks/24 weeks	I, A
	Ledipasvir/sofosbuvir for patients who are non-black, HIV uninfected, without cirrhosis, and whose HCV RNA < 6 million IU/mL	8 weeks	I, B
	Daclatasvir/sofosbuvir	12 weeks/24 weeks	I, B/IIa, B
	Simeprevir/sofosbuvir ± ribavirin	12 weeks/24 weeks	II, B
Genotype 1b without/with compensated cirrhosis	Ledipasvir/sofosbuvir	12 weeks	I, A
	Velpatasvir/sofosbuvir	12 weeks	I, A
	Grazoprevir/elbasvir	12 weeks	I, A
	Paritaprevir/ritonavir/ombitasvir/dasabuvir	12 weeks	I, A
	Ledipasvir/sofosbuvir for patients who are non-black, HIV uninfected, without cirrhosis, and whose HCV RNA < 6 million IU/mL	8 weeks	I, B
	Daclatasvir/sofosbuvir	12 weeks/24 weeks	I, B/IIa, B
	Simeprevir/sofosbuvir	12 weeks/24 weeks	II, B
Genotype 2 without/with compensated cirrhosis	Velpatasvir/sofosbuvir	12 weeks	I, A
	Sofosbuvir + weight-based RBV	12 weeks/16 weeks	I, A/IIb, C
	Daclatasvir/sofosbuvir	12 weeks/16–24 weeks	IIa, B
Genotype 3 without/with compensated cirrhosis	Velpatasvir/sofosbuvir	12 weeks	I, A
	Daclatasvir/sofosbuvir ± ribavirin	12 weeks/24 weeks	I, A/IIa, B
	Sofosbuvir + weight-based RBV + weekly peg-IFN	12 weeks	I, A
	Sofosbuvir + weight-based RBV	24 weeks	I, B
Genotype 4 without/with compensated cirrhosis	Ledipasvir/sofosbuvir	12 weeks	I, A/IIa, B
	Velpatasvir/sofosbuvir	12 weeks	I, A
	Grazoprevir/elbasvir	12 weeks	I, A/IIa, B
	Paritaprevir/ritonavir/ombitasvir + weight-based RBV	12 weeks	I, B
	Sofosbuvir + weight-based RBV	24 weeks	IIa, B
	Sofosbuvir + weight-based RBV + weekly peg-IFN	12 weeks	II, B
	Simeprevir/sofosbuvir	12 weeks	II, B
	Daclatasvir/sofosbuvir	12 weeks	II, B
Genotype 5 and 6 with and without cirrhosis	Velpatasvir/sofosbuvir	12 weeks	I, B
	Daclatasvir/sofosbuvir	12 weeks	I, B
	Ledipasvir/sofosbuvir	12 weeks	IIa, B
	Sofosbuvir + weight-based RBV + weekly peg-IFN	12 weeks	IIa, B

**Table 2:** Shimoyama classification defining four adult T-cell leukemia/lymphoma subtypes.

	Smoldering	Chronic	Lymphoma	Acute
Lymphocyte count (×10^3^/L)	<4	≥4	<4	High
Flower cells (%)	<5	≥5	≤1	High
LDH level	≤1.5 times ULN	<2.5 times ULN	High	High
Ca^2+^ level	Normal	Normal	High	High
Skin and/or lung involvement	+/−	+/−	+/−	+/−
Lymph node involvement	No	+/−	Yes	+/−
Spleen/liver involvement	No	+/−	+/−	+/−
Central nervous system/bone/pleural/ascites	No	No	+−	+/−

Reproduced with permission from Marcais, A., Therapeutic Options for Adult T-Cell Leukemia/Lymphoma; published by *Curr. Oncol. Rep.*, 2013. ULN, upper limit of normal.
